# Assessing the cost-effectiveness of COVID-19 vaccines in a low incidence and low mortality setting: the case of Thailand at start of the pandemic

**DOI:** 10.1007/s10198-022-01505-2

**Published:** 2022-08-11

**Authors:** Yi Wang, Nantasit Luangasanatip, Wirichada Pan–ngum, Wanrudee Isaranuwatchai, Juthamas Prawjaeng, Sompob Saralamba, Christopher Painter, Jamaica Roanne Briones, Yot Teerawattananon

**Affiliations:** 1 Saw Swee Hock School of Public Health, National University of Singapore, Singapore, Singapore; 2 Mahidol-Oxford Tropical Medicine Research Unit (MORU), Faculty of Tropical Medicine, Mahidol University, Bangkok, Thailand; 3 Health Intervention and Technology Assessment Program (HITAP), Ministry of Public Health, Nonthaburi, Thailand

**Keywords:** SARS-CoV-2, COVID-19, Vaccine, Vaccination, Cost-effectiveness, Economic evaluation, Thailand

## Abstract

**Objective:**

This study aimed to assess the cost-effectiveness of COVID-19 vaccines, preferred COVID-19 vaccine profiles, and the preferred vaccination strategies in Thailand.

**Methods:**

An age-structured transmission dynamic model was developed based on key local data to evaluate economic consequences, including cost and health outcome in terms of life-years (LYs) saved. We considered COVID-19 vaccines with different profiles and different vaccination strategies such as vaccinating elderly age groups (over 65s) or high-incidence groups, i.e. adults between 20 and 39 years old who have contributed to more than 60% of total COVID-19 cases in the country thus far. Analyses employed a societal perspective in a 1-year time horizon using a cost-effectiveness threshold of 160,000 THB per LY saved. Deterministic and probabilistic sensitivity analyses were performed to identify and characterize uncertainty in the model.

**Results:**

COVID-19 vaccines that block infection combined with social distancing were cost-saving regardless of the target population compared to social distancing alone (with no vaccination). For vaccines that block infection, the preferred (cost-effective) strategy was to vaccinate the high incidence group. Meanwhile, COVID-19 vaccines that reduces severity (including hospitalization and mortality) were cost-effective when the elderly were vaccinated, while vaccinating the high-incidence group was not cost-effective with this vaccine type. Regardless of vaccine type, higher vaccination coverage, higher efficacy, and longer protection duration were always preferred. More so, vaccination with social distancing measures was always preferred to strategies without social distancing. Quarantine-related costs were a major cost component affecting the cost-effectiveness of COVID-19 vaccines.

**Conclusion:**

COVID-19 vaccines are good value for money even in a relatively low-incidence and low-mortality setting such as Thailand, if the appropriate groups are vaccinated. The preferred vaccination strategies depend on the type of vaccine efficacy. Social distancing measures should accompany a vaccination strategy.

**Supplementary Information:**

The online version contains supplementary material available at 10.1007/s10198-022-01505-2.

## Introduction

The COVID-19 pandemic will not end until the global roll-out of vaccines is fully realized and when people from all corners of the world are directly or indirectly protected against the virus. Although national health regulators around the globe have authorized emergency use of COVID-19 vaccines at a very fast pace, having licensed vaccines is not enough to achieve global control of COVID-19 [1]. The vaccines need to be produced at scale, priced affordably and delivered strategically so that local outbreaks can be controlled, and life can return to normal.

Currently, there are more than a hundred COVID-19 vaccine candidates in clinical studies, with 15 approved for human use [2]. Approved vaccines differ substantially in costs, cold-storage requirements, clinical efficacy, and safety profile, and vaccines in development will likely differ further. As such, country governments face decisions on choosing the most appropriate vaccine(s) and will continue to do so – given their country profiles, outbreak situation, and vaccination strategy. Such questions governments face include, for example: (i) what are the most desirable COVID-19 vaccine characteristics, e.g. alternative types of vaccine efficacy, protection duration and price, for prevention and control from an economic perspective; (ii) which subpopulation groups – stratified by age, occupation, geographical location or other characteristics – should be prioritized given limited vaccine supplies; (iii) what is the best value for money in terms of a combination of non-pharmacological interventions (NPIs) and vaccines for controlling COVID-19; (iv) what is the economically justifiable price in negotiations for a vaccine.

Economic evaluation can be usefully employed to address such above questions, by systematically comparing the relative costs and consequences of different vaccination strategies. This type of analysis is very relevant to high-income and upper-middle-income countries, which will need to pay for the full cost of COVID-19 vaccines. They should be well-prepared for short and long-term procurement plans, including vaccine selection and pricing negotiations, to ensure that the significant investment in a COVID-19 vaccine justifies its opportunity costs from displaced investment in the health sector or across public programs.

This paper shares the methodological approach and results from an economic evaluation conducted to inform policy decisions regarding COVID-19 vaccination in Thailand. Thailand implemented Universal Health Coverage Scheme in 2002 [3]. Thailand’s healthcare system includes both public and private sectors, where the public health insurance schemes are offered through three different schemes: (1) the Universal Coverage Scheme (covering approximately 80% of the population); (2) the Social Security Scheme (13% of the population); and (3) the Civil Servant Medical Benefit Scheme (7% of the population) [4]. Unlike traditional economic evaluations, which inform about accepting or rejecting a vaccine, this Thai government commissioned study addresses different policy questions faced by a country with a relatively low local transmission and mortality (the situation for Thailand in January–May, 2020; more details on number of COVID-19 cases and deaths during this time are shown in Supplementary Information 1). For example, Thailand closed its border on March 26, 2020, which refers to both international and national travel. Working from home, social distance, and curfew was implemented at similar period. These non-pharmaceutical interventions were relaxed towards the end of June of 2020. Due to the early implementation of these interventions, the incidence rate and mortality rate of COVID-19 remained low in Thailand in 2020. There was a surge in the number of cases from towards the end of December 2020 due to the arrival of Delta variant.

While the interventions at the early stage were effective in containing COVID-19, these interventions also imposed economic losses as well as inconvenience to people’s daily life. COVID-19 vaccine was considered as the solution to concur COVID-19; however, COVID-19 vaccines did not arrive in Thailand until February 2021 [5]. In addition, the tourism sector is a vital part of the Thai economy, contributing to around 12% of the national gross domestic product [6]. As such, the Thai government needs to decide which type of vaccines and whom to vaccinate so that future large outbreaks of COVID-19 can be avoided. Subsequently, exploring whether costly NPIs, such as social distancing and international travel restrictions, can be eased or adjusted to allow the economy to return to the pre-COVID-19 level. These analyses were conducted in 2021 at a time when the precise characteristics of vaccination and their cost-effectiveness were more uncertain, tourism/international travel was very low due to travel restrictions and involved a lengthy quarantine period, and the expected supply of vaccines was much lower than was realized (affecting the policy-making process). Therefore, vaccine prioritization was an important healthcare decision for the Thai government. This study further assists the Thai government in making transparent and fair recommendations on initially prioritized population groups for vaccination, and subsequent rounds of vaccination.

## Methods

### Transmission dynamic and economic model

An age-structured epidemiological model developed by the Covid-19 International Modelling Consortium (CoMo consortium) was adapted by Luangasanatip N et al. [7, 8] to estimate age-stratified incidence and deaths of COVID-19 infection under different scenarios of vaccine characteristics, vaccination strategy, and combination of NPIs. In brief, the transmission model was developed based on an SEIR (Susceptible-Exposed-Infectious-Recovered) structure. The I compartment of the transmission dynamic model was stratified by the severity of infection (no symptoms, mild symptoms, intensive care unit and intensive care unit with a ventilator). Country-specific epidemiology, clinical, vaccine and NPI parameters were used in the model. Different conditions of hypothetical vaccine characteristics were considered based on the WHO target product profile (TPP), [9] including different target populations (high risk elderly aged ≥ 65 years or a high-incidence group aged 20–39 years), vaccinated population sizes (5 million, 9 million and 15 million people vaccinated (regardless of number of doses)), efficacy type (infection blocking and severity reduction which aim to reduce both hospitalization and mortality), level of efficacy (50, 70 and 90%), duration of protection (0.5 and 1 year), and social distancing (SD) implementation (with and without SD) were explored to estimate the impact of a COVID-19 vaccine on incidence and death. In the study setting (Thailand), there are approximately 12 million elderly (~ 18% of total population of approximately 66 million people) and those aged 15–59 years accounted for 64% of total population as of January 2022. More details on the transmission dynamic model are available in Supplementary Information 2. The outputs from the transmission dynamic model were fed into the economic model for evaluating economic consequences (cost and health outcomes or life-years, LYs, saved) of various vaccination strategies (based on target population, level of efficacy, and duration of protection) compared to no vaccination with social distancing. The study adopted a societal perspective using a one-year time horizon The study did not require the approval of the independent ethics committee because it was based on a mathematical and economic model to simulate the cost and outcome and did not include human participants.

### Model input parameters

Although Thailand has methodological guidelines for conducting economic evaluations of healthcare interventions, it was agreed that certain aspects of these guidelines were not appropriate for evaluations of COVID-19 vaccines during stakeholder engagement meetings. This issue was discussed further in a related methodological principles research article for the Thai and Singaporean economic evaluations of COVID-19 vaccination [10].

#### Cost variables

The cost analysis was done from a societal perspective. Cost parameters included direct medical costs, direct non-medical costs and indirect costs. *Direct medical costs* for COVID-19 infection treatment were retrieved from electronic individual records of the e-claim database of the National Health Security Office (NHSO) [11]. Due to limited data availability, the treatment cost for asymptomatic cases was assumed to be zero, on the basis that these cases would not be identified by the healthcare system and therefore would not be treated. Costs for contact tracing and testing included the cost of real-time reverse transcription polymerase chain reaction (RT-PCR), personal protective equipment (PPE) for technicians [12], and costs of tracing derived from literature [13]. The proportion of asymptomatic cases detected which were traced and tested was obtained from a systematic review and meta-analysis [14]. The number of patients under investigation (PUI) per confirmed case was derived from data from the Thai Ministry of Public Health (MOPH) and was pegged at 44.4 persons [15]. Costs for transmission prevention including face masks and disinfection agents were obtained from an e-commerce market, which has been a major supplier during the pandemic [16]. Costs for the vaccination program, including vaccine price (for two doses), supply chain, and logistic and hospital-based administration were based on publicly available data and existing literature from a flu vaccination program in Thailand, respectively [17, 18]. Costs of treating thromboembolic events, a serious adverse event (AE), were analyzed by identifying ICD-10 codes related to thromboembolic events [19] from an e-claim database of the NHSO in Thailand [20]. The incidence rate of thromboembolic events was obtained from existing evidence [19]. *Direct non-medical costs* related to quarantine costs were obtained from the Department of Disease Control (DDC) [21], including accommodation and meals for 14 days. Based on personal communication with DDC staff, we assumed 10 high-risk of infection PUI per confirmed case. *Indirect costs* from implementing non-pharmaceutical interventions (NPIs) including the impact of social distancing on the economy was estimated using gross domestic product [22], the stock market index [23], and other control variables to control for local and global COVID-19 cases, global economic status, and other government policies. Details of the time series analysis conducted can be found in the Supplementary Information 6 and a related methodological research article [24]. All cost parameters were converted to 2020 values using the consumer price index (CPI) [25] and presented in Thai baht (THB) where 1 USD = 35.8 THB (Table 1).

**Table 1:** Parameter values

Variable name	Variable Explanation	Mean	SD	Source
Cost_dmed_nsev_1	Direct medical cost per patient for patient aged 4 and below with mild to moderate symptoms	32,893	23,379	(11), a
Cost_dmed_nsev_2	Direct medical cost per patient for patient aged 5 to 14 with mild to moderate symptoms	29,139	20,254	(11), a
Cost_dmed_nsev_3	Direct medical cost per patient for patient aged 15 to 39 with mild to moderate symptoms	33,457	22,245	(11), a
Cost_dmed_nsev_4	Direct medical cost per patient for patient 40 to 64 with mild to moderate symptoms	37,514	23,661	(11), a
Cost_dmed_nsev_5	Direct medical cost per patient for patient aged 65 and above with mild to moderate symptoms	45,435	26,174	(11), a
Cost_dmed_sev_1	Direct medical cost per patient for patient aged 4 and below with severe symptoms	32,893	23,379	(11), a
Cost_dmed_sev_2	Direct medical cost per patient for patient aged 5 to 14 with severe symptoms	29,139	20,254	(11), a
Cost_dmed_sev_3	Direct medical cost per patient for patient aged 15 to 39 with severe symptoms	89,030	17,710	(11), a
Cost_dmed_sev_4	Direct medical cost per patient for patient aged 40 to 64 with severe symptoms	102,028	58,977	(11), a
Cost_dmed_sev_5	Direct medical cost per patient for patient aged 65 and above with severe symptoms	104,306	64,199	(11), a
Cost_dmed_dead	Direct medical cost per patient for patient that were infected with COVID-19 and died	84,066	49,295	(11), a
Cost_dmed_asyp	Direct medical cost per patient for asymptomatic patient	0	NA	Assumption
Cost_screen	Cost of COVID-19 screening	1500	375	(12)
Cost_vac_sup	Cost of vaccine supply management per dose	19.59	4.8975	(18)
Cost_vac_admin	Cost of vaccination administration at hospital per dose	124.03	31.0075	(18)
Cost_vac_aqui	Cost of vaccine acquisition per dose	93	23.25	(17)
Cost_mask_year	Cost of mask per person per year	365	91.25	(16)
Cost_hygiene_year	Cost of hygiene and sanitizing per person per year	1200	300	(16)
Cost_trace	Cost of contract tracing per person traced	450	112.5	(13)
Cost_quarantine	Cost of quarantine at designated facilities	15,000	3750	(21)
Cost_npi_sd	Cost of social distancing per year	4.55E + 10	2.28E + 10	(24), a
Num_test_asyoversym	Number of asymptomatic cases tested per symptomatic case	0.18	0.046	(14)
Num_trace_perdetect	Number of people traced per detected case	44.35	18	(15)
Num_quanrantine_perdetect	Number of people quarantined per detected case	10	2.5	Assumption
Adverse_inci	Incidence of adverse event	6E-6	1.5E-6	(19)
Adverse_cost	Direct medical cost for patients with adverse event	23,968	9093	(20), a

All cost parameters were converted into 2020 Thai Baht

*a* Analysis done by authors

#### Outcome estimates

Outcomes were measured in LY saved by using the age-stratified life loss, using the Thailand standard lifetable [26].

### Data analysis

The primary results of the analysis were incremental cost-effectiveness ratios (ICERs), in THB per LY saved. We used the ceiling threshold of 160,000 THB per LY saved in our analysis, as recommended by the Subcommittee for the Development of the National List of Essential Medicines (NLEM).

Sensitivity analyses were performed to account for parameter uncertainty. A one-way deterministic sensitivity analysis (DSA) was conducted by varying one parameter at a time, while other parameters remain unchanged, to understand the impact of each individual parameter on the net monetary benefit (NMB) using a ceiling threshold of 160,000 THB per LY saved. We focused on the parameters listed in Table 1. We selected several scenarios examining the impact of different parameters on the incremental NMB comparing vaccination strategies with highest NMBs to no vaccine and comparing vaccination strategies with highest NMBs to alternative strategies. Social distancing was assumed to be implemented in all the scenarios in the sensitivity analysis. The results of the DSA were presented using tornado plots. A probabilistic sensitivity analysis (PSA) was conducted by sampling all model parameters from their statistical distributions. A value of information (VOI) analysis was conducted based on the results of the PSA.

## Results

### Cost-effectiveness analysis

**Fig. 1:**
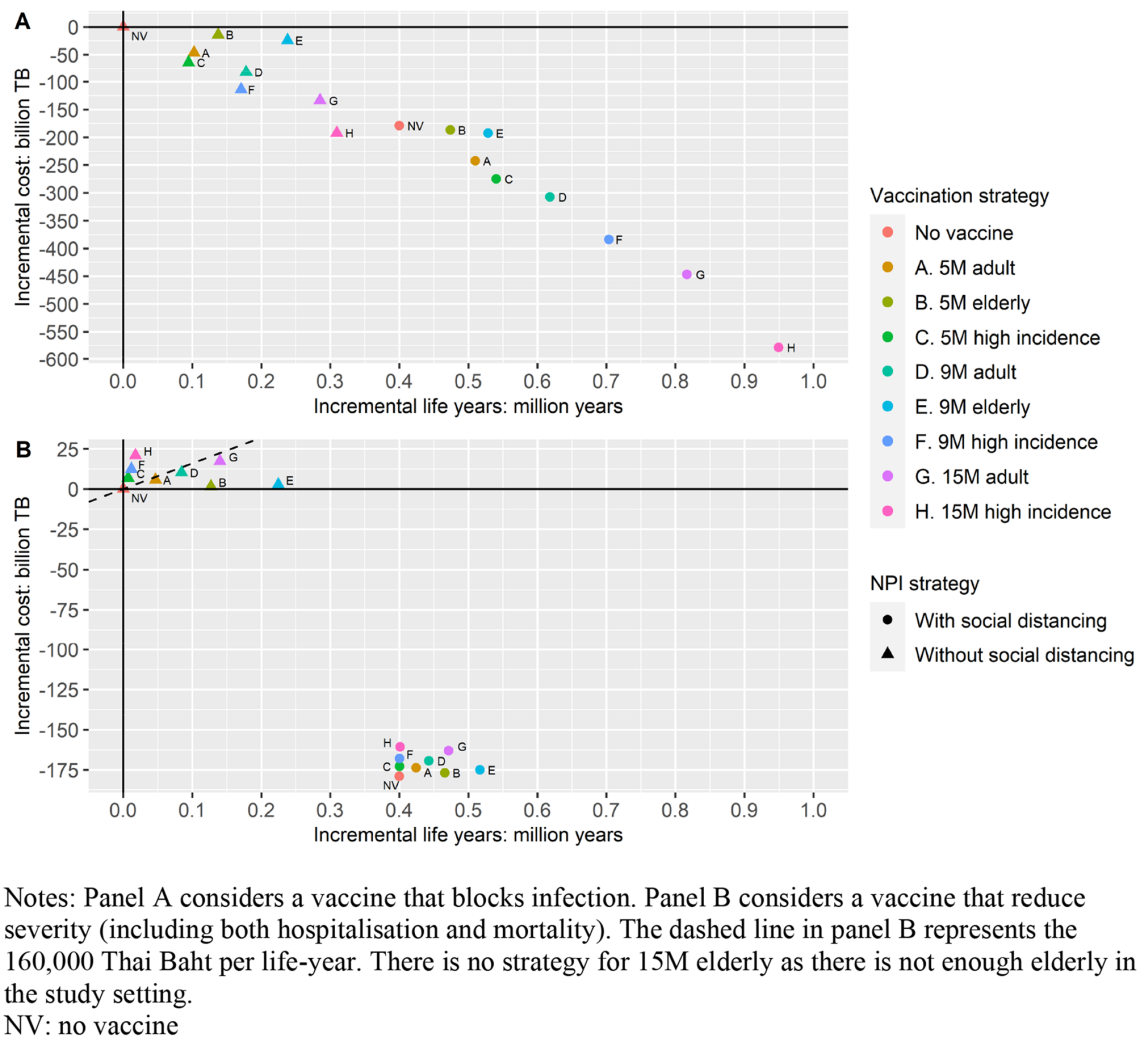
Cost-effectiveness plane – Different Vaccination Strategies for blocking infection and reducing severity

Figure 1 shows the cost-effectiveness plane results when a vaccine with 70% efficacy and 1-year protection duration was considered. The comparator was no vaccine and without social distancing located at the origin. Panel A shows the results for a vaccine that blocks infection; panel B shows the results for a vaccine that reduces severity. If the vaccine blocks infection, under the scenario without social distancing, all vaccination strategies are cost-saving compared to no vaccination. Comparing vaccinating high-incidence group to vaccinating the elderly, e.g. triangle F versus triangle E, vaccinating high-incidence group saved more cost but fewer life years. Vaccinating high-incidence group was preferred over vaccinating elderly using the ceiling cost-effectiveness threshold of 160,000 THB per QALY (according to the Thai National HTA Guideline). Social distancing alone without vaccination, denoted by the dotted no vaccine, was more cost-saving compared to vaccination without social distancing when the number of vaccine was limited. When social distancing was implemented, all vaccination strategies were cost-saving compared to no vaccination, though high-incidence groups should be prioritized over general adults and the elderly. A figure using the strategy of no vaccine combining with social distancing as the comparator is presented in Supplementary Information 3 Fig S4 Panel A.

If the vaccine reduces severity, under the scenario without social distancing, vaccinating the elderly and the general adults were cost-effective. Vaccinating the elderly was preferred over vaccinating adults with lower incremental cost and more life years saved, e.g. triangle E versus triangle D. Social distancing alone provided better value for money compared to vaccination, i.e. dotted no vaccine versus triangles. When social distancing was implemented, only vaccinating the elderly represented good value for money. A figure using the strategy of no vaccine combining with social distancing as the comparator is presented in Supplementary Information 3 Fig S4 Panel B.

Breakdown costs for selected scenarios are presented in Table 2. While the results are not equivalent to budget impact analysis, they show the yearly cost or yearly budget required for each component if the Thailand government decides to fully subsidize the cost. Costs of quarantine, direct medical cost, and cost of contact tracing are lower with social distancing compared to without social distancing. Cost of quarantine and cost of contact tracing are lower with vaccines that blocks infection compared to vaccines that reduces severity.

**Table 2:** Breakdown cost (in Billion 2020 Thai Baht)

Scenario	Dmed	Mask	Hygiene	Vaccine.aqui	Vaccine.supchain	Vaccine.admin	Adv.Event	CT	Quarantine	SD	Total
Baseline_w_sd_40_rv1	107.02	7.65	25.15	0	0	0	0	51.16	384.48	45.51	620.98
Vac_Eff1_5M_AgeEld_70_sd40_rv1	104.65	7.65	25.15	0.93	0.20	1.24	0.0007	50.25	377.67	45.51	613.26
Vac_Eff1_5M_AgeHig_70_sd40_rv1	87.60	7.65	25.15	0.93	0.20	1.24	0.0007	41.93	315.11	45.51	525.32
Vac_Eff1_09M_AgeEld_70_sd40_rv1	102.89	7.65	25.15	1.67	0.35	2.23	0.0013	49.57	372.59	45.51	607.62
Vac_Eff1_09M_AgeHig_70_sd40_rv1	65.64	7.65	25.16	1.67	0.35	2.23	0.0013	31.46	236.44	45.51	416.12
Vac_Eff1_15M_AgeHig_70_sd40_rv1	26.70	7.65	25.16	2.79	0.59	3.72	0.0022	12.83	96.41	45.51	221.36
Vac_Eff3_5M_AgeEld_70_sd40_rv1	106.84	7.65	25.15	0.93	0.20	1.24	0.0007	51.17	384.57	45.51	623.26
Vac_Eff3_5M_AgeHig_70_sd40_rv1	107.57	7.65	25.15	0.93	0.20	1.24	0.0007	51.54	387.37	45.51	627.16
Vac_Eff3_09M_AgeEld_70_sd40_rv1	106.70	7.65	25.15	1.67	0.35	2.23	0.0013	51.18	384.64	45.51	625.09
Vac_Eff3_09M_AgeHig_70_sd40_rv1	108.00	7.65	25.15	1.67	0.35	2.23	0.0013	51.84	389.65	45.51	632.07
Vac_Eff3_15M_AgeHig_70_sd40_rv1	108.63	7.65	25.15	2.79	0.59	3.72	0.0022	52.29	393.05	45.51	639.39
Baseline_w_sd_00_rv1_full	151.19	7.65	25.15	0	0	0	0	72.35	543.79	0.00	800.13
Vac_Eff1_5M_AgeEld_70_sd0_rv1	147.28	7.65	25.15	0.93	0.20	1.24	0.0007	70.89	532.81	0.00	786.14
Vac_Eff1_5M_AgeHig_70_sd0_rv1	137.90	7.65	25.15	0.93	0.20	1.24	0.0007	66.08	496.68	0.00	735.83
Vac_Eff1_09M_AgeEld_70_sd0_rv1	144.39	7.65	25.15	1.67	0.35	2.23	0.0013	69.81	524.71	0.00	775.97
Vac_Eff1_09M_AgeHig_70_sd0_rv1	127.64	7.65	25.15	1.67	0.35	2.23	0.0013	61.24	460.27	0.00	686.22
Vac_Eff1_15M_AgeHig_70_sd0_rv1	111.49	7.65	25.15	2.79	0.59	3.72	0.0022	53.60	402.82	0.00	607.81
Vac_Eff3_5M_AgeEld_70_sd0_rv1	150.70	7.65	25.15	0.93	0.20	1.24	0.0007	72.30	543.43	0.00	801.61
Vac_Eff3_5M_AgeHig_70_sd0_rv1	151.75	7.65	25.15	0.93	0.20	1.24	0.0007	72.81	547.25	0.00	806.98
Vac_Eff3_09M_AgeEld_70_sd0_rv1	150.33	7.65	25.15	1.67	0.35	2.23	0.0013	72.27	543.14	0.00	802.80
Vac_Eff3_09M_AgeHig_70_sd0_rv1	152.22	7.65	25.15	1.67	0.35	2.23	0.0013	73.19	550.08	0.00	812.54
Vac_Eff3_15M_AgeAdu_70_sd0_rv1	152.28	7.65	25.15	2.79	0.59	3.72	0.0022	73.41	551.73	0.00	817.33

This table shows the breakdown cost for each component. All cost were converted into 2020 Thai Baht

*Dmed* direct medical cost, *Vaccine.aqui* cost of vaccine acquisition, *Vaccine.supchain* cost of vaccine supply chain, *Vaccine.admin* cost of vaccine administration, *Adv.Event* direct medical cost due to vaccine-related adverse event, *CT* contract tracing, *SD* social distancing, *Baseline_w_sd_40_rv1* no vaccine and with social distancing, *Baseline_w_sd_00_rv1* no vaccine and without social distancing, *Scenarios start with Eff* vaccine efficacy type + number of people being vaccinated + population group to vaccinate + vaccine efficacy + with/without social distancing + protection duration

Vaccine efficacy type: *Eff1* infection blocking, *Eff3* severity reduction

Number of people being vaccinated: *5 M* 5 million, *09 M* 9 million, *15 M* 15 million

Population group to vaccinate: *AgeEld* elderly group, *AgeHig* high-incidence group

Vaccine efficacy: 70 – 70%

With/without social distancing: *sd40* with social distancing, *sd0* without social distancing

Protection duration: *rv1* 1 year

Of the considered cost components, the leading cost was due to quarantine, which contributed approximately 30–70% of the total policy costs. Vaccine-related costs, including vaccine acquisition, vaccine supply chain, vaccine administration, and medical costs for vaccine-related adverse events, were relatively small compared to, for example, contact tracing and COVID-19 treatment costs.

**Fig. 2:**
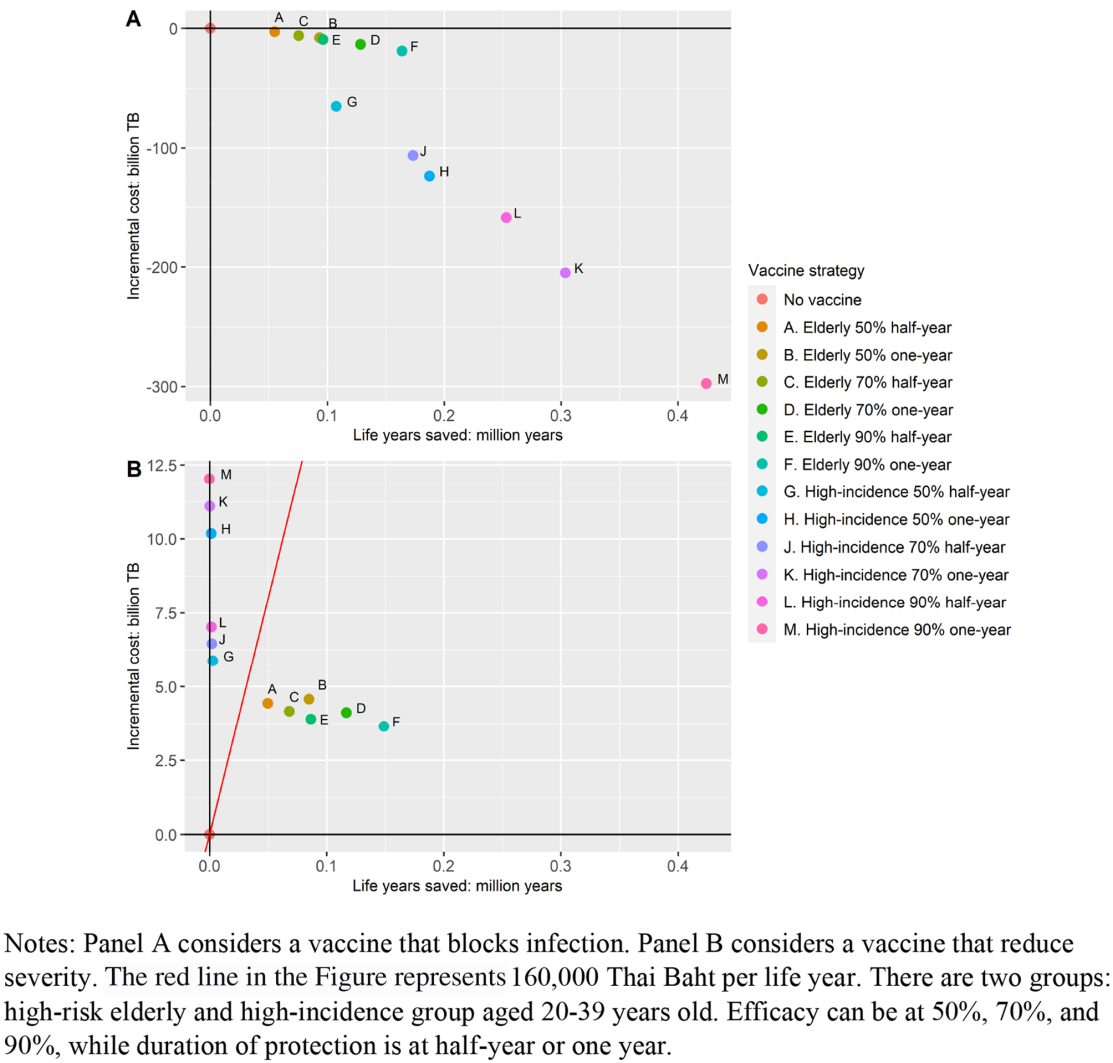
Cost-effectiveness Plane – Different Vaccine Profiles for blocking infection and reducing severity

Figure 2 shows the cost-effectiveness plane, where different vaccine profiles and vaccination strategies were considered. All scenarios considered included social distancing and a vaccinated population size of nine million. The figure also shows that the high-incidence group should be prioritized over the elderly if vaccines block infection, while the elderly should be prioritized if the vaccine only reduces severity. This figure also illustrates that vaccines with higher efficacy and longer protection duration are cost-effective compared to no vaccination with social distancing. COVID-19 vaccines with one-year protection duration and 20% lower efficacy represent better value for money than vaccines with 6 months protection duration and 20% higher efficacy, suggesting that duration of protection is an important vaccine characteristic. Results comparing different vaccine profiles and vaccination strategies without social distancing are presented in Supplementary Information 3 Fig S5. The results show the similar patterns as the results with social distancing.

Results exploring the impact of using different ceiling thresholds are presented in Supplementary Information 3 Fig S6. If vaccines block infection, all vaccination strategies yield positive NMBs at any ceiling threshold compared to no-vaccine scenario. Nevertheless, if vaccines reduce severity, offering vaccines to the elderly yields positive NMBs at ceiling thresholds higher than 20,000–100,000 THB per LY depending on levels of vaccine efficacy and protection duration.

### Sensitivity analysis

**Fig. 3:**
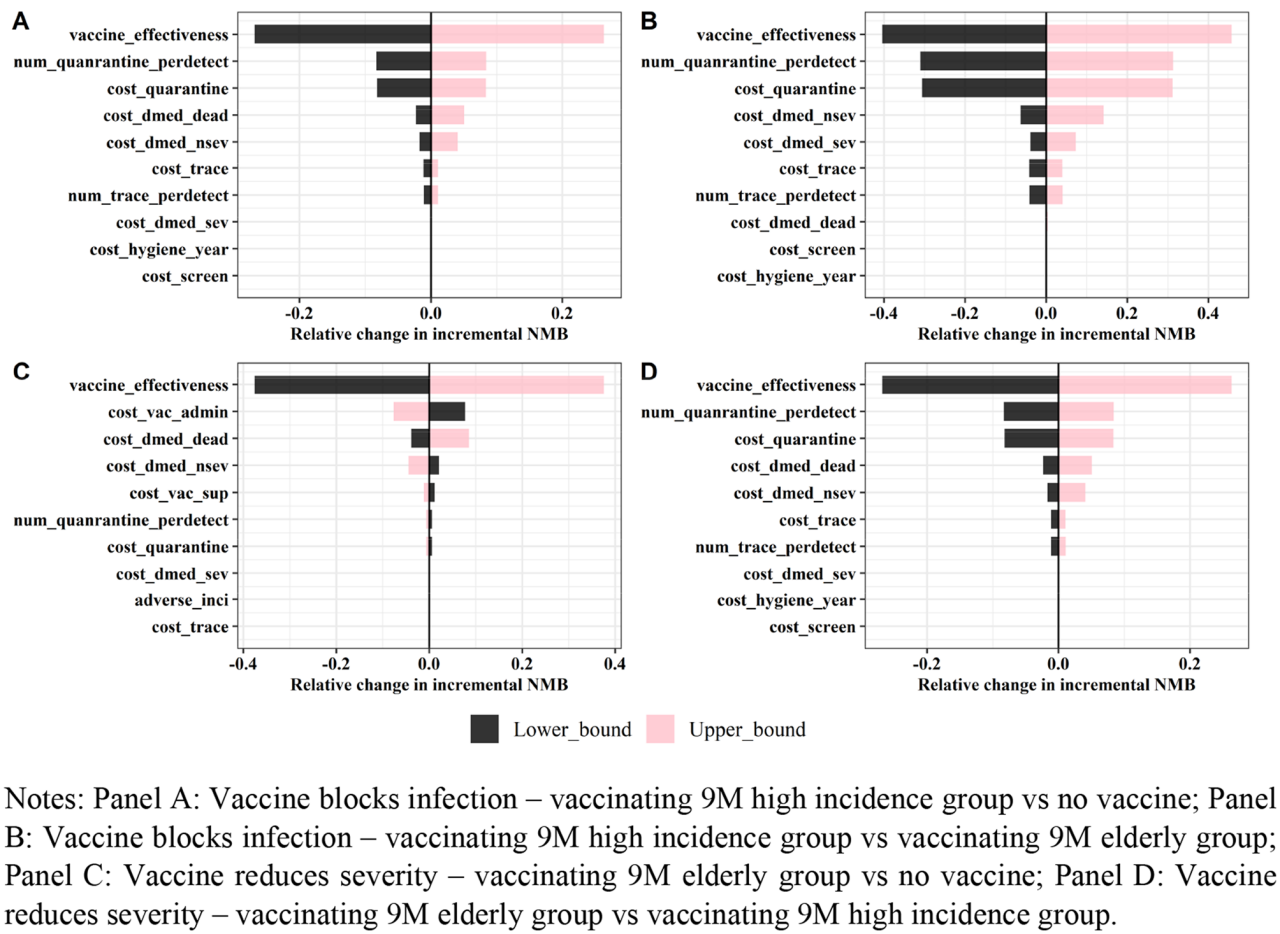
Deterministic Sensitivity Analysis

Figure 3 shows the results of the one-way DSA under four different vaccine and target population scenarios. A vaccine with 70% efficacy and 1-year protection duration was considered. The x-axis represents the relative change in incremental NMB. A larger value indicates the parameter has higher impact on the incremental NMB. The cost-effectiveness results from the model are most sensitive to the vaccine efficacy parameter. Vaccine supply and administration costs did not have a large effect on the model results except when vaccines block infection. The model was also sensitive to costs related to quarantine for PUI when considering vaccines that block transmission.

**Fig. 4:**
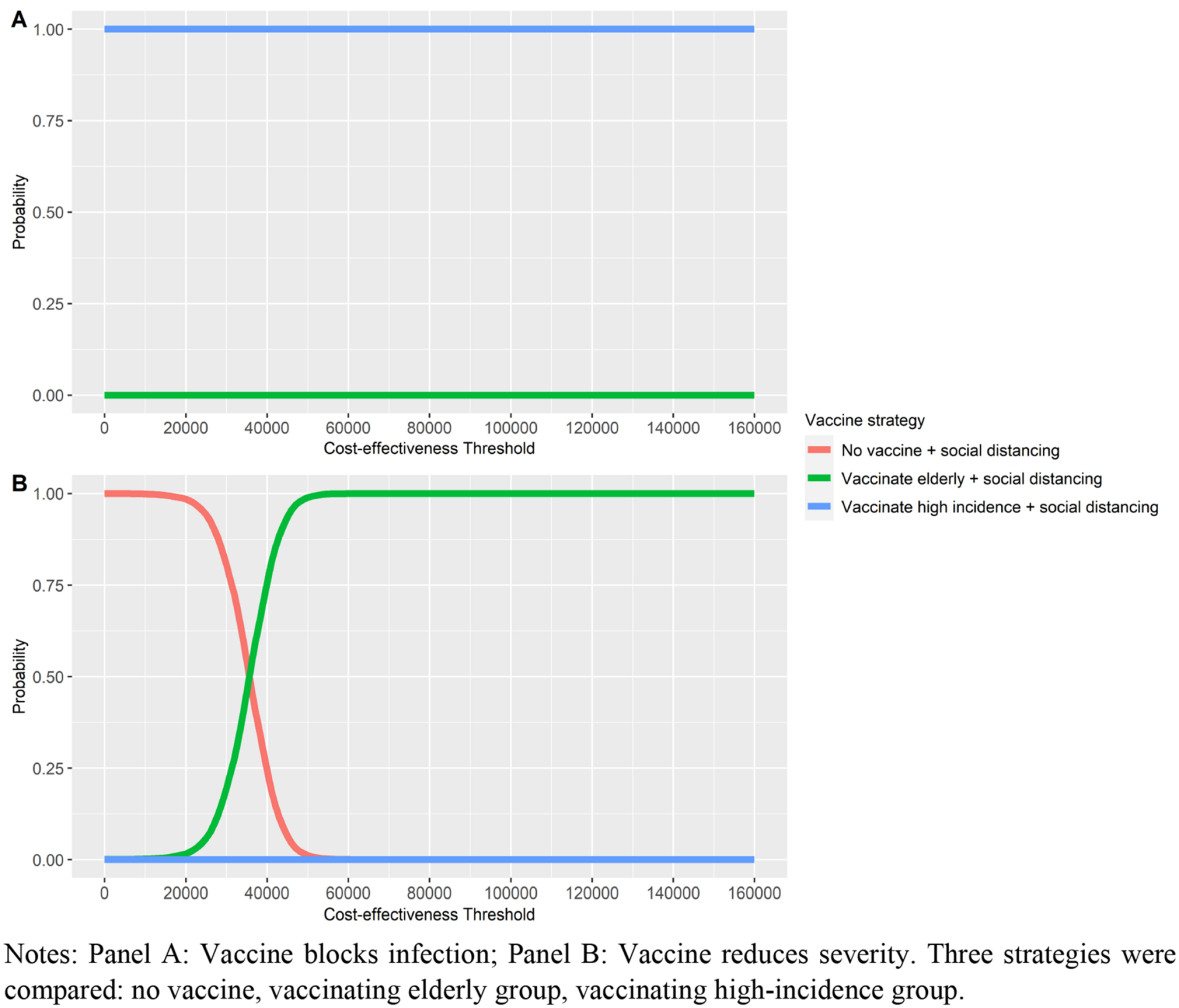
Probabilistic Sensitivity Analysis

Focusing on the strategies with social distancing, three strategies were compared in the PSA, including no vaccine, vaccinating elderly, and vaccinating high-incidence group. The PSA results, in Fig. 4 Panel A, show that the probability of vaccinating high-incidence groups with vaccines that block infection being the most cost-effective strategy was equal to 1 at all ceiling thresholds. For vaccines that reduce severity, in Fig. 4 Panel B, the probability of vaccinating high-incidence groups being the most cost-effective was equal to 0 at all ceiling thresholds. The probability of vaccinating elderly groups being the most cost-effective strategy was 1 for ceiling thresholds larger than 60,000 THB per LY. Hence, at a ceiling threshold of 160,000 THB per LY, the uncertainties in the preferred policy mainly arose from the type of vaccine efficacy considered.

**Fig. 5:**
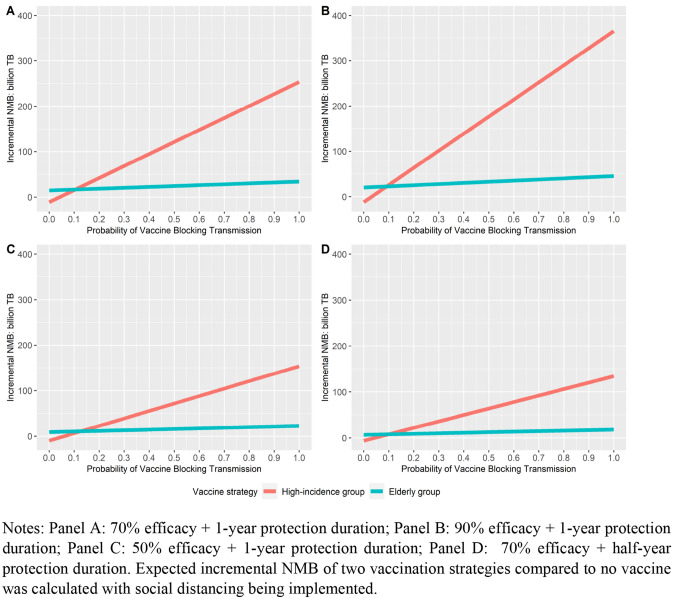
Expected incremental NMB vs Probability of Vaccine Blocking Transmission

As the actual profile of the vaccine, e.g. vaccine type, efficacy, protection duration, were unknown to the policymakers at the time point when they need to make decisions, we explored the expected incremental NMB of vaccinating high-incidence groups and vaccinating elderly groups with vaccines with different probability in blocking infection (*p*) and probability of reductions in severity (1 – *p*) at a fixed level of overall efficacy. We assumed that the vaccine will either block infection for the vaccinated population or reduce severity for the vaccinated population. The expected incremental NMB was calculated as a linear combination of the incremental NMB of the scenario with the vaccine blocking infection and incremental NMB of the scenario with the vaccine reducing severity. Specifically, as shown in Fig. 5, we considered four scenarios with combinations of different overall vaccine efficacy and protection duration. The results inform that only an approximate 10–20% probability of blocking infection is enough to justify prioritizing high-incidence groups over elderly groups for COVID-19 vaccination in Thailand.

### VOI analysis

**Fig. 6:**
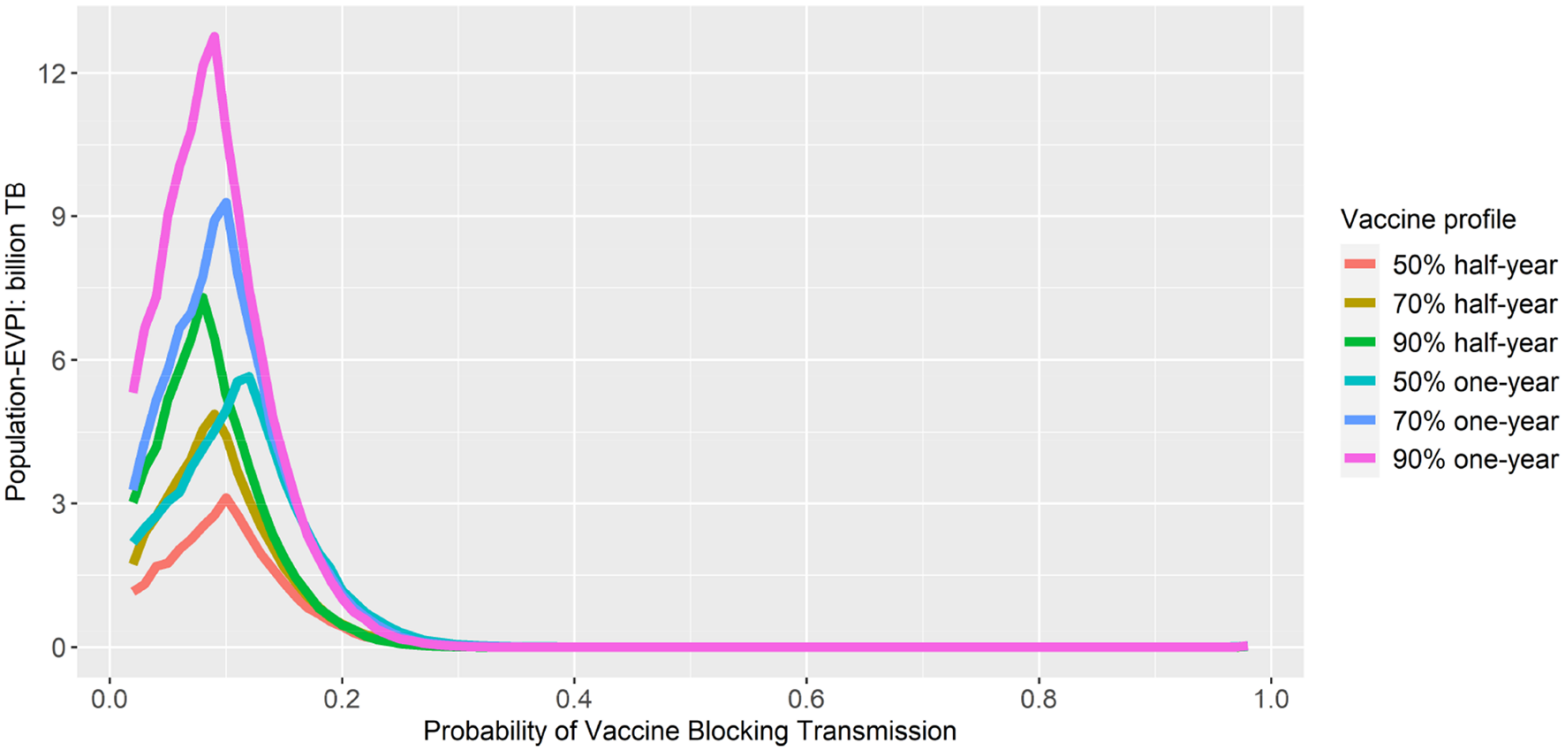
EVPI vs Probability of vaccine blocking infection

While an approximate 10–20% probability of blocking infection is enough to justify prioritizing high-incidence groups over elderly groups, there was no preliminary information available about the probability of vaccines blocking infection. Hence, we examined the population level expected value of perfect information (EVPI) by considering a range of probabilities that vaccines can block infection using a ceiling threshold of 160,000 THB per LY, as shown in Fig. 6. If the prior belief that the probability of the vaccine blocking infection is high enough, e.g. greater than 0.4, the EVPI, or the benefit of doing additional research to reduce the uncertainty in parameters, was close to 0. From this point onward, it is preferred to vaccinate high-incidence groups without requiring additional research to reduce parameter uncertainties. The peaks of EVPI were around 10–20%. This finding supported the results from Fig. 5 where the expected benefits are similar between vaccinating high-incidence group and elderly group. Under these scenarios, additional research can be justified to reduce the uncertainties in parameters.

## Discussion

This study is the first economic evaluation of COVID-19 vaccines to inform policy decisions in an upper middle-income country with relatively low incidence and low mortality. From a societal perspective, if vaccines block infection, then vaccines are cost-saving for all population groups compared to no vaccination. If vaccines would only reduce severity, then vaccinating elderly groups is the preferred strategy. Although these findings indicate that cost-effectiveness of vaccination strategies depends on vaccine type; all vaccine efficacy trials, except AZD1222 (Oxford/AstraZeneca) trials [27], were not designed to address the key question of whether vaccines can reduce transmission [28]. However, observational studies conducted in Israel [29], England [30], and the United States [31] demonstrated relatively high vaccine effectiveness against SARS-CoV-2 B.1.1.7 or Alpha variant transmission (approximately 50–70%) in real-world situations. These empirical studies provide reassurance that, at the very least, mRNA and AstraZeneca COVID-19 vaccines offer dual benefits in terms of protecting symptomatic and severe cases as well as preventing infections after getting the recommended doses of the vaccines. Applying this evidence to our study supports that vaccines can provide better value for money, meaning that vaccinating high-incidence groups is likely to be the preferred strategy in the Thai setting. With new COVID-19 variants keeping emerging over time, protection of COVID-19 vaccine is also expected to change [32, 33]. A contingent vaccination plan, considering the vaccine efficacy type, efficacy level, and protection duration in response to the changing epidemiological conditions, is important for all governments to adjust vaccination strategy and priority timely.

This local study applied a framework for "collaborative modelling for effective policy implementation and evaluation" through iterative stakeholder participation and discussion in order to enhance trust, accountability, and public ownership to decisions [34]. Series of public and stakeholder consultation meetings were conducted throughout the study. Results of this study were deliberated in the public media [35, 36] and the Vaccine Management Subcommittee including the Vaccine Research Working Group under the Subcommittee, a part of the Thai Ministry of Public Health [37]. The findings were used to guide vaccination strategies in Thailand where high-incidence populations were one of the priority groups, especially in areas with active local transmission. As of June 2, 2021, 3.7 million people (about 4% of the total Thai population) had received at least one dose of either AstraZeneca or CoronaVac vaccines. Of these, about 55% were health professionals or frontline civil servants, 35% were from high-incidence groups and 10% were elderly or people with underlying health conditions. Before the arrival of delta and omicron variants, this vaccination policy may be more appropriate for Thailand as the country had low case fatality (0.67%) compared to other countries, especially in Europe and America with case fatality ranging between 1 and 12% [38]. In addition, many resource-rich countries anticipate relaxing social distancing (or other NPIs) upon vaccination roll-out since they can procure large supplies of vaccines, meaning that they can expect to achieve high levels of vaccine coverage. Given the limited vaccine supply, our study suggests social distancing should remain a vital part of COVID-19 response measures in Thailand. Table 2 illustrates the breakeven cost of social distancing that makes social distancing policy a cost-effective option, which estimated to be about 1% of GDP depending on the vaccination strategy.

Our results are in line with the previous findings in the United Kingdom (UK) [39], the United States (US) [40] and Turkey [41] that COVID-19 vaccines are either cost-saving or represent good value for money in high-incidence settings. Nevertheless, none of the previously mentioned studies consider restricted vaccine supplies which is a major issue faced by many governments in resources-limited settings. Only the study in the US examined the impact of delayed vaccine supplies by a few months [40]. As such, they did not fully assess the trade-off between sub-population groups within the same setting. Furthermore, only the UK study explored the value of NPIs alongside vaccination, it also came to the same conclusion as this study that social distancing practice should be maintained until population-wide vaccine coverage is sufficient to achieve herd immunity [39]. Also, our study and the UK study [39] agree that serious adverse events do not impact the value for money conclusion for vaccines. Unlike other studies, our findings indicate that mandatory state quarantine for PUIs or infected individuals consume significant costs to the Thai society. This is part of the NPIs for which the costs were fully subsidized by the Thai government. In addition, it is uncertain whether differential quarantine and testing policies for vaccinated populations who have had high-risk exposure to COVID-19 patients can be implemented since there is currently a lack of evidence, policies and guidelines [42].

The results of this study must be interpreted with caution. Notably, the mathematical model was matched to the projected data of Thai population’s contact patterns [43] and national COVID-19 outbreak data. Thus, the findings are likely to be primarily applicable to similar settings with limited or low-level community transmission. Specifically, the context and findings were from the mid-2020 period in Thailand which may appear outdated given the COVID-19 situation in Thailand in 2021 and 2022. However, the findings may inform future pandemic and situations in countries that face similar contexts where procurement of vaccines might already take place, but the continuing supply was uncertain. Our economic evaluation was able to give some insights on the affordability of the new strategy (i.e. the “can we do it” question). The results provided justification for the government to secure additional budget from external sources (loan money) in order to procure COVID-19 vaccines (in order to complement or even substitute NPIs). However, our economic evaluation does have little power in explaining the accessibility to implement (the “do we reach those in need” question), or the measurement of the adequacy of the strategy developed (the “monitoring” question); areas where future research can explore further [44–46].

Moreover, the vaccines considered in the analyses were hypothetical, based on the WHO’s TPP characteristics. The results of this study cannot directly correspond to individual vaccines currently available in the global market. We selected this approach because it allows decision-makers assessing the value for money of new vaccines, or existing vaccines as new information becomes available. Although this study adopted a societal perspective, we did not include the productivity loss due to COVID-19 infected patients. This will not alter our findings and conclusion given the small size of people infected in the country. Also, this study considered relatively low costs of social distancing, when compared to the UK study which applied a much higher societal cost for NPIs (1% compared to 2% of GDP) [39]. We also acknowledge that this is a difficult question with no clear-cut answers, as country contexts can change rapidly, and there is also no standard methodology to accurately quantify the economic cost of NPIs. Lastly, there is a lot of individual parameter uncertainty in the model, particularly related to vaccine-related parameters (e.g. vaccine hesitancy), COVID-19 clinical parameters, and economic parameters. These uncertainties should be addressed by evidence generation activities from effective monitoring and evaluation systems. For example, the effectiveness and efficiency of vaccination program will depend significantly on vaccine hesitancy in the population, leading to an important research area on vaccine hesitancy where more details in the Thai context can be found elsewhere [44, 45, 47]. As such, inputs from this study could be used to inform and develop these frameworks for monitoring and evaluation of COVID-19 vaccination including its short- and long-term impact at national and global levels.

## Conclusion

COVID-19 vaccines are likely to be cost-saving or cost-effective in resource-limited settings with limited or low-level community transmission. Regarding the target population, the preferred vaccination strategy depended on the type of vaccine efficacy realized. High-incidence groups should be vaccinated if the probability of vaccines blocking infection is high. Elderly groups should be prioritized over high-incidence groups if the only effect of vaccines is to prevent severe infections among COVID-19 cases.

## Supplementary Information

Below is the link to the electronic supplementary material.

Supplementary file1 (DOCX 21 KB)

Supplementary file2 (DOCX 1847 KB)

## Abbreviations

NPIs: Non-pharmacological interventions
CoMo consortium: The COVID-19 International Modelling Consortium
SEIR: Susceptible–Exposed–Infectious–Recovered
TPP: Target product profile
SD: Social distancing
Lys: Life-years
NHSO: National Health Security Office
RT-PCR: Real-time reverse transcription polymerase chain reaction
PPE: Personal protective equipment
PUI: Patients under investigation
MOPH: Ministry of Public Health
AE: Adverse event
DDC: Department of Disease Control
THB: Thai baht
ICERs: Incremental cost-effectiveness ratios
NLEM: National List of Essential Medicines
PSA: Probabilistic sensitivity analysis
DSA: Deterministic sensitivity analysis
NMB: Net monetary benefit
VOI: Value of information
EVPI: Expected value of perfect information
AZD1222: Oxford/AstraZeneca
