# Supplementary material for: Assessing the cost-effectiveness of COVID-19 vaccines in a low incidence and low mortality setting: the case of Thailand at start of the pandemic
Source: Eur J Health Econ. 2022 Aug 11;24(5):735–48. doi: 10.1007/s10198-022-01505-2 (PMC9366779; doi:10.1007/s10198-022-01505-2)
Supplement: Supplementary file 2 — Supplementary file2 (DOCX 1847 KB) [file 10198_2022_1505_MOESM2_ESM.docx]

# Maximizing value for money of COVID-19 vaccines in a low incidence and low mortality setting

Yi Wang^1,^, Nantasit Luangasanatip^2^, Wirichada Pan–ngum^2^, Wanrudee Isaranuwatchai^3*^, Juthamas Prawjaeng^3^, Sompob Saralamba^2^, Christopher Painter^3^, Jamaica Roanne Briones^1^, Yot Teerawattananon^1,3^

^1^ Saw Swee Hock School of Public Health, National University of Singapore, Singapore

^2^ Mahidol-Oxford Tropical Medicine Research Unit (MORU), Faculty of Tropical Medicine, Mahidol University, Thailand.

^3^ Health Intervention and Technology Assessment Program (HITAP), Ministry of Public Health, Thailand.

* Corresponding author

E-mail: wanrudee.i@hitap.net

**Supplementary Information 1: Number of COVID-19 reported cases and deaths from 1 January 2020 up to 10^th^ May 2020 (Thailand’s first outbreak)**

| date | Number of reported cases per day | Number of deaths per day |
| --- | --- | --- |
| 31/12/2019 | 0 | 0 |
| 1/1/2020 | 0 | 0 |
| 2/1/2020 | 0 | 0 |
| 3/1/2020 | 0 | 0 |
| 4/1/2020 | 0 | 0 |
| 5/1/2020 | 0 | 0 |
| 6/1/2020 | 0 | 0 |
| 7/1/2020 | 0 | 0 |
| 8/1/2020 | 0 | 0 |
| 9/1/2020 | 0 | 0 |
| 10/1/2020 | 0 | 0 |
| 11/1/2020 | 0 | 0 |
| 12/1/2020 | 1 | 0 |
| 13/1/2020 | 0 | 0 |
| 14/1/2020 | 0 | 0 |
| 15/1/2020 | 0 | 0 |
| 16/1/2020 | 0 | 0 |
| 17/1/2020 | 1 | 0 |
| 18/1/2020 | 0 | 0 |
| 19/1/2020 | 0 | 0 |
| 20/1/2020 | 0 | 0 |
| 21/1/2020 | 0 | 0 |
| 22/1/2020 | 2 | 0 |
| 23/1/2020 | 0 | 0 |
| 24/1/2020 | 1 | 0 |
| 25/1/2020 | 1 | 0 |
| 26/1/2020 | 2 | 0 |
| 27/1/2020 | 0 | 0 |
| 28/1/2020 | 6 | 0 |
| 29/1/2020 | 0 | 0 |
| 30/1/2020 | 0 | 0 |
| 31/1/2020 | 5 | 0 |
| 1/2/2020 | 0 | 0 |
| 2/2/2020 | 0 | 0 |
| 3/2/2020 | 0 | 0 |
| 4/2/2020 | 6 | 0 |
| 5/2/2020 | 0 | 0 |
| 6/2/2020 | 0 | 0 |
| 7/2/2020 | 0 | 0 |
| 8/2/2020 | 7 | 0 |
| 9/2/2020 | 0 | 0 |
| 10/2/2020 | 0 | 0 |
| 11/2/2020 | 1 | 0 |
| 12/2/2020 | 0 | 0 |
| 13/2/2020 | 0 | 0 |
| 14/2/2020 | 0 | 0 |
| 15/2/2020 | 1 | 0 |
| 16/2/2020 | 0 | 0 |
| 17/2/2020 | 1 | 0 |
| 18/2/2020 | 0 | 0 |
| 19/2/2020 | 0 | 0 |
| 20/2/2020 | 0 | 0 |
| 21/2/2020 | 0 | 0 |
| 22/2/2020 | 0 | 0 |
| 23/2/2020 | 0 | 0 |
| 24/2/2020 | 0 | 0 |
| 25/2/2020 | 2 | 0 |
| 26/2/2020 | 3 | 0 |
| 27/2/2020 | 0 | 0 |
| 28/2/2020 | 1 | 0 |
| 29/2/2020 | 1 | 0 |
| 1/3/2020 | 0 | 1 |
| 2/3/2020 | 1 | 0 |
| 3/3/2020 | 0 | 0 |
| 4/3/2020 | 0 | 0 |
| 5/3/2020 | 4 | 0 |
| 6/3/2020 | 1 | 0 |
| 7/3/2020 | 2 | 0 |
| 8/3/2020 | 0 | 0 |
| 9/3/2020 | 0 | 0 |
| 10/3/2020 | 3 | 0 |
| 11/3/2020 | 6 | 0 |
| 12/3/2020 | 13 | 0 |
| 13/3/2020 | 3 | 0 |
| 14/3/2020 | 7 | 0 |
| 15/3/2020 | 32 | 0 |
| 16/3/2020 | 33 | 0 |
| 17/3/2020 | 30 | 0 |
| 18/3/2020 | 35 | 0 |
| 19/3/2020 | 60 | 0 |
| 20/3/2020 | 50 | 0 |
| 21/3/2020 | 89 | 0 |
| 22/3/2020 | 188 | 0 |
| 23/3/2020 | 122 | 0 |
| 24/3/2020 | 106 | 3 |
| 25/3/2020 | 107 | 0 |
| 26/3/2020 | 111 | 0 |
| 27/3/2020 | 91 | 1 |
| 28/3/2020 | 108 | 1 |
| 29/3/2020 | 141 | 1 |
| 30/3/2020 | 137 | 2 |
| 31/3/2020 | 128 | 1 |
| 1/4/2020 | 121 | 2 |
| 2/4/2020 | 104 | 3 |
| 3/4/2020 | 103 | 4 |
| 4/4/2020 | 89 | 1 |
| 5/4/2020 | 102 | 3 |
| 6/4/2020 | 51 | 3 |
| 7/4/2020 | 38 | 1 |
| 8/4/2020 | 111 | 3 |
| 9/4/2020 | 54 | 2 |
| 10/4/2020 | 50 | 1 |
| 11/4/2020 | 45 | 2 |
| 12/4/2020 | 33 | 3 |
| 13/4/2020 | 28 | 2 |
| 14/4/2020 | 34 | 1 |
| 15/4/2020 | 30 | 2 |
| 16/4/2020 | 29 | 3 |
| 17/4/2020 | 28 | 1 |
| 18/4/2020 | 33 | 0 |
| 19/4/2020 | 32 | 0 |
| 20/4/2020 | 27 | 0 |
| 21/4/2020 | 19 | 1 |
| 22/4/2020 | 15 | 1 |
| 23/4/2020 | 13 | 1 |
| 24/4/2020 | 15 | 0 |
| 25/4/2020 | 53 | 1 |
| 26/4/2020 | 15 | 0 |
| 27/4/2020 | 9 | 1 |
| 28/4/2020 | 7 | 2 |
| 29/4/2020 | 9 | 0 |
| 30/4/2020 | 7 | 0 |
| 1/5/2020 | 6 | 0 |
| 2/5/2020 | 6 | 0 |
| 3/5/2020 | 3 | 0 |
| 4/5/2020 | 18 | 0 |
| 5/5/2020 | 1 | 0 |
| 6/5/2020 | 1 | 1 |
| 7/5/2020 | 3 | 0 |
| 8/5/2020 | 8 | 0 |
| 9/5/2020 | 4 | 1 |
| 10/5/2020 | 5 | 0 |

**Supplementary Information 2: Details on dynamic model**

## Modelling structure

In this study, we adapted an opensource compartmental age-structured model which is based on the SEIR (Susceptible-Exposed-Infective-Recovered) structure developed by the COVID-19 International Modelling Consortium (CoMo consortium) (1-3). In the model, the infected compartments were stratified by symptoms, severity and treatment seeking and access. There are four sub-compartments accounting for different severities of COVID-19 infection which are i) asymptomatic, ii) mild to moderate symptomatic, iii) ICU and iv) ICU with ventilator.

**Fig. S1 Model structure**

## Model parameters and outputs

The model uses publicly available country-specific data to define the population structure (4) and mixing contact patterns (5) as model inputs. The model also uses publicly available country-specific data on cases and mortality (6) for visual calibration of model parameters to user-selected baseline scenarios. In this study the model was calibrated against the data from the first wave of the pandemic in Thailand. In the calibration, the reported daily new cases and deaths of COVID-19 infection were obtained from the government’s online data, which assumes that 100% of all cases were identified and reported (7). We believe that this assumption is reasonable as the first outbreak was mainly due to 3 clusters that occurred during the middle of March and there were several months of no cases in Thailand after the initial outbreak and no excess mortality (6).

One other key set of parameters stratified by age was hospitalisation rates (AHR) and in-hospital fatality rates (IFR), which were derived from previous published information from Wuhan, China and available local data from Thailand from the 55 deaths up to 6^th^ June 2020, respectively (7). Virus-related parameters including the incubation period, duration of symptomatic infection, risk of asymptomatic infection, symptomatic infection (having clinical symptoms), admittance to ICU with and without a ventilator were adopted from the values used in the CoMo model (8-10). Given the gap in knowledge on immunity acquired post-infection, we assumed that natural immunity lasts an average of two years (1, 2). The admission information for those with hospitalisation including the percentage of patients requiring ICU/ventilator, length of stay, and probability of death given ICU/ventilator were derived from published data (11-13). We used an 18 month time horizon for all model simulations, which allows for a duration of 12 months post-vaccination. The primary model outputs were the number of COVID-19 cases by severity, as well as deaths.

## Non-pharmaceutical interventions (NPIs)

As the model calibration was based on the pandemic of the first wave, NPIs applied in Thailand included hand hygiene and masking, social distancing, travel ban (or border closure) were tracked and incorporated in the model (Figure and Table below). As test, trace and isolation (TTI) varied spatially and temporally, it was accounted for in the system dynamics via the force of infection, which changed over time. The infection rate and effective coverage of each NPI including hand hygiene and social distancing was estimated from visual calibration, by comparing the reported daily incidence and total cases and deaths against the model prediction when the time for introduction of each NPI was clearly stated. The baseline of 30% effective hand hygiene means reduction in the effective contact with infected people by 30% while 40% social distancing means reduction in number of daily contacts across age groups by 40%. The set of estimated parameters given the optimal fit to the surveillance data from the first outbreak during January to 6^th^ June 2020 was used in the baseline scenario.

**Fig. S2 Observed reported cases with the tracked timeline of non-pharmaceutical interventions implemented during the first outbreak in Thailand**


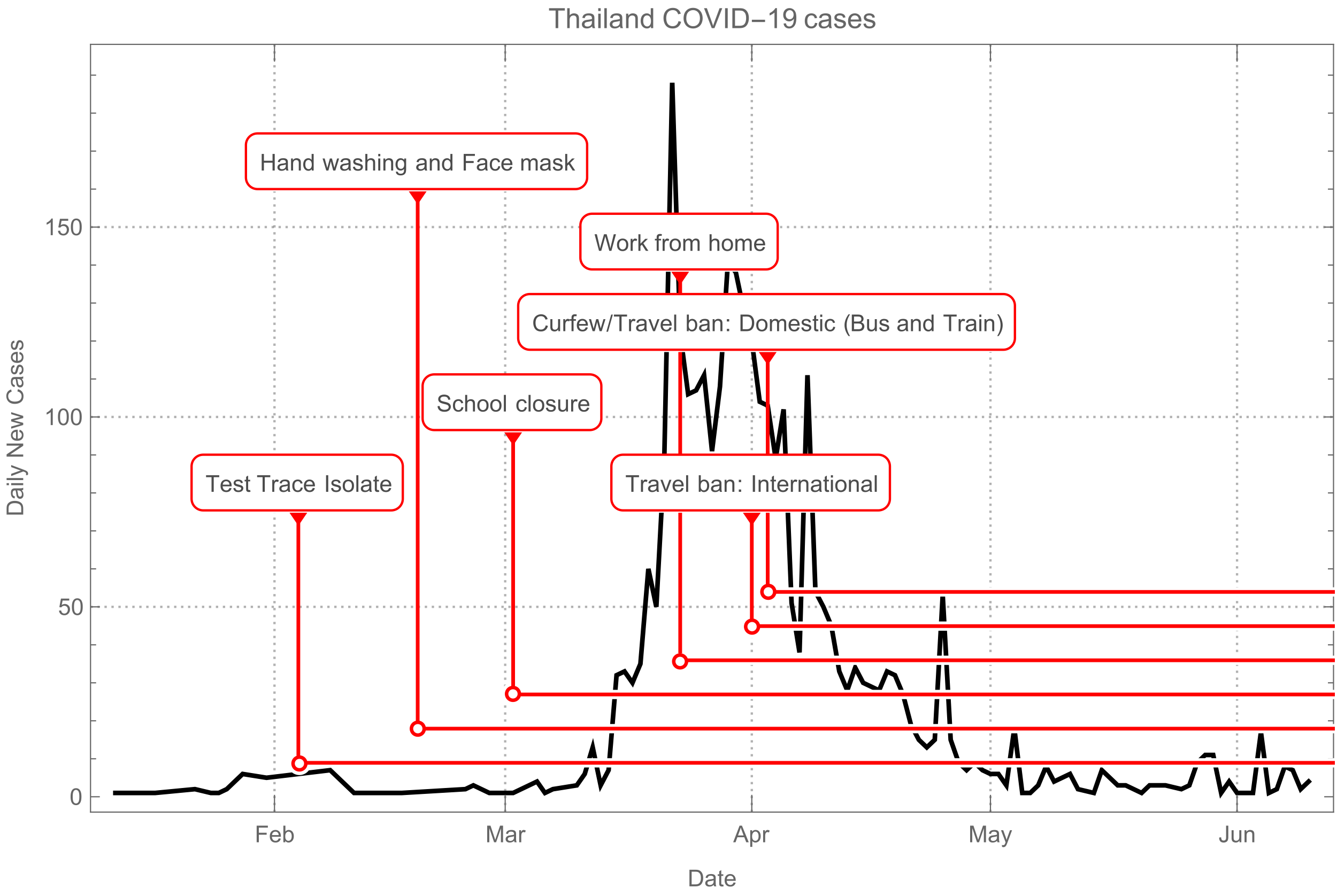


**Table S1. Details of Non-Pharmaceutical Interventions and Vaccination Implementation used in the model**

| **Intervention** | **Start date** | **Stop date** | **Duration** | **Variable** | **Values** | **Note** |
| --- | --- | --- | --- | --- | --- | --- |
| 1. Hand hygiene and Face mask | 1 Jan 21 | 31 Mar 22 | 1 year 3 months | Effective Coverage | 30% | Model calibration |
| 2. Travel ban release | 1 July 21 | 31 Mar 22 | 9 months | Number of imported cases (per day) | 100 | Assumption |
| 3. Social distance | 1 July 21 | 31 Mar 22 | 9 months | Effective Coverage | 40% | Model calibration |
| 4. Vaccination | 1 Apr 21 | 31 May 21 | 2-month uptake rate | Effectiveness and Coverage | vary | Assumption |

**Fig. S3 Visual calibration results to estimate the efficacy of several implemented non-pharmaceutical interventions during the first outbreak.**


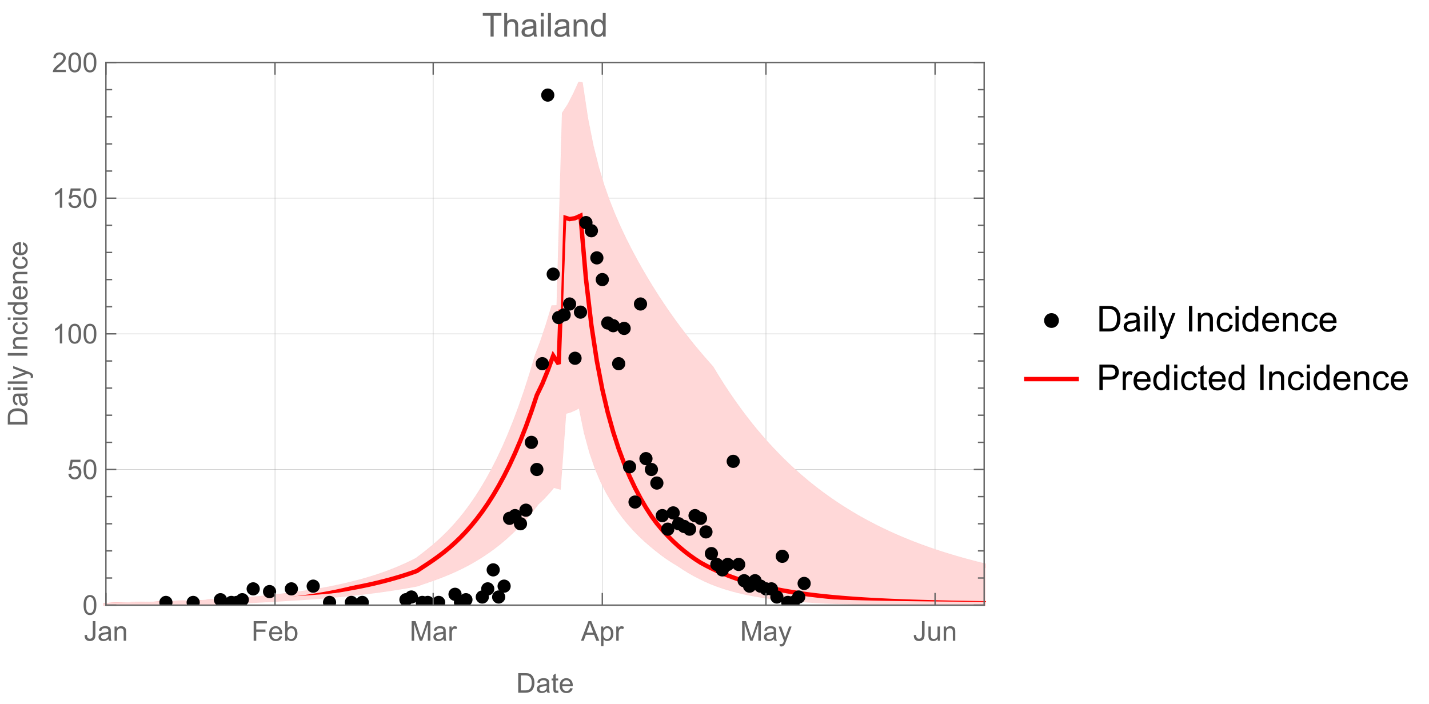


## Vaccine characteristics and implementation

The introduction of COVID-19 vaccination was expanded from the original CoMo model version 15.0, where we defined the vaccine efficacy by 3 characteristics; i) reduced susceptibility (Efficacy 1), ii) reduced transmissibility (Efficacy 2) and iii) reduced severity including mortality (Efficacy 3). Reduced susceptibility (Efficacy 1) means direct reduction in infection rate among vaccinated group while reduced transmissibility (Efficacy 2) means reduction in force of infection among infections of vaccinated group and reduced severity including mortality (Efficacy 3) means reduced symptoms and progression to hospitalisation among those with vaccination. We assumed that only one vaccine was used at a time, with a one dose regimen. The supply of vaccines is likely to be limited for Thailand, we therefore assumed that it would be approximately 9 million doses in 2021. The assumed vaccine supply approximately corresponds to the size of the smallest potential group for vaccination, the high-risk of severe symptoms group (over 65 years old), who account for approximately 9 million people in Thailand. This assumed vaccine supply allowed reasonable comparison between vaccinating this group and the high incidence group (aged 20 to 39 years). This age range was based on incidence data of wave 1 in Thailand which can be different from situation in 2021 and 2022. It is further assumed that given the limited doses of vaccines, the Thai healthcare system would be capable of delivering the vaccine to reach the target coverage within two months. As the protective duration of all the vaccines available is still unknown, we assumed two scenarios, a short-term and long-term duration of protection of 0.5 or 1 year, respectively. In the base case, a duration of protection of 1 year was assumed.

## Base case scenario

Prior to the availability of the vaccine, there was assumed to be a small amount of local transmission in the country spread from an original case upon day 1 in the model and no imported cases. To evaluate the value of the vaccine, we assumed that following the roll-out of the vaccine there would be an accompanying relaxation of travel restrictions and quarantine measures to allow imported cases to enter the model. It was assumed that 100 cases of COVID-19 would be imported per day, based on expert opinion from policymakers and representatives from the chamber of commerce. We assumed a constant coverage of effective hand hygiene and facemask wearing of 30% among the population based on our model visual calibration of the first outbreak. Social distancing measures were applied, which reduced social contacts by 40% (excluding those at home, work and school). More details can be found at <https://www.researchsquare.com/article/rs-270635/v1>.

**Supplementary Information 3**

**Fig. S4 Cost-effectiveness Plane – Different Vaccination Strategies for blocking infection and reducing severity: using the strategy of no vaccine but with social distancing as the comparator**

**
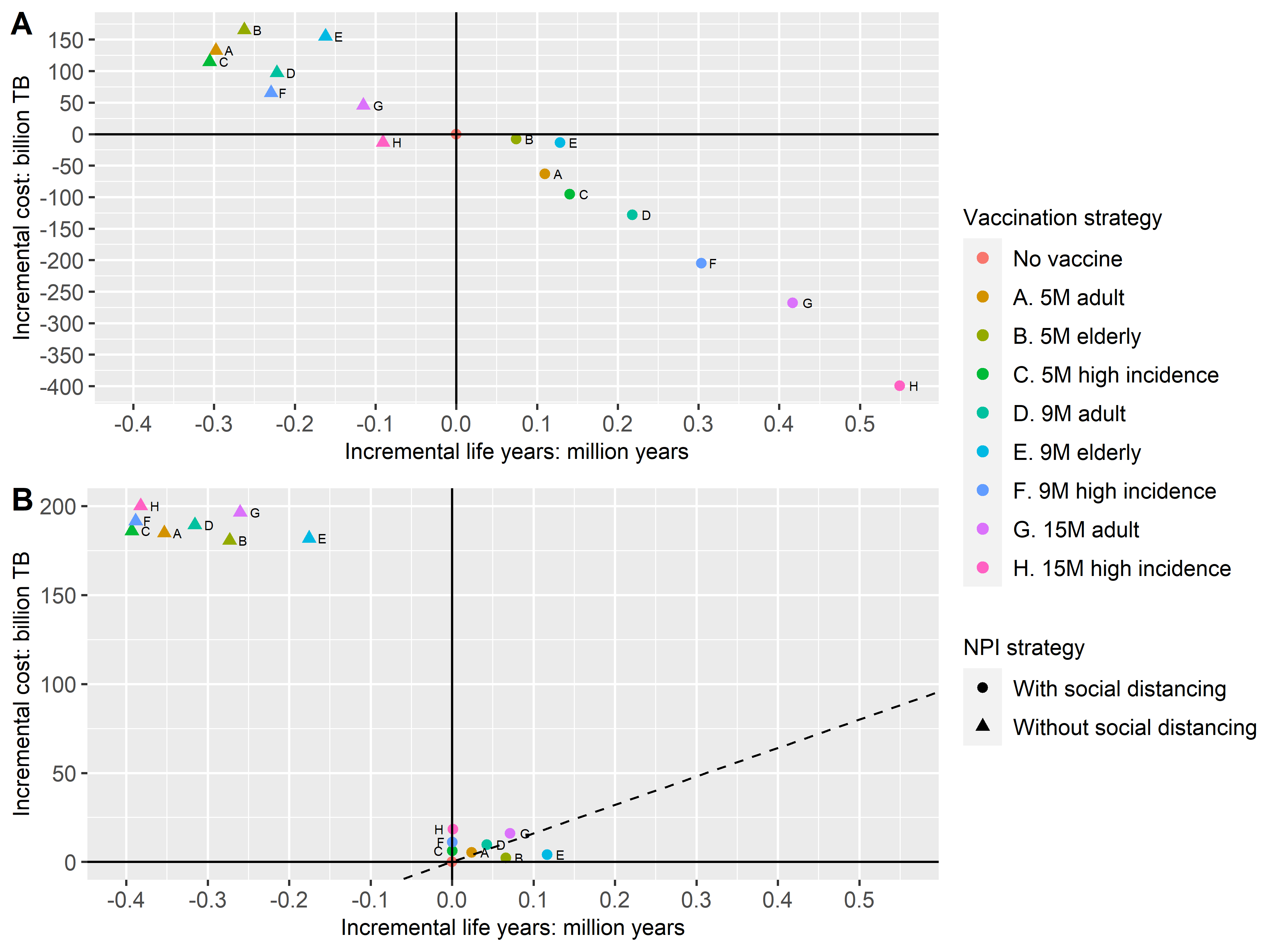
**

Notes: Panel A considers a vaccine that blocks infection. Panel B considers a vaccine that reduce severity (including both hospitalisation and mortality). The dashed line in panel B represents the 160,000 Thai Baht per life-year. There is no strategy for 15M elderly as there is not enough elderly in the study setting.

**Fig. S5 Cost-effectiveness Plane – Different Vaccine Profiles for blocking infection and reducing severity: scenarios without social distancing**

**
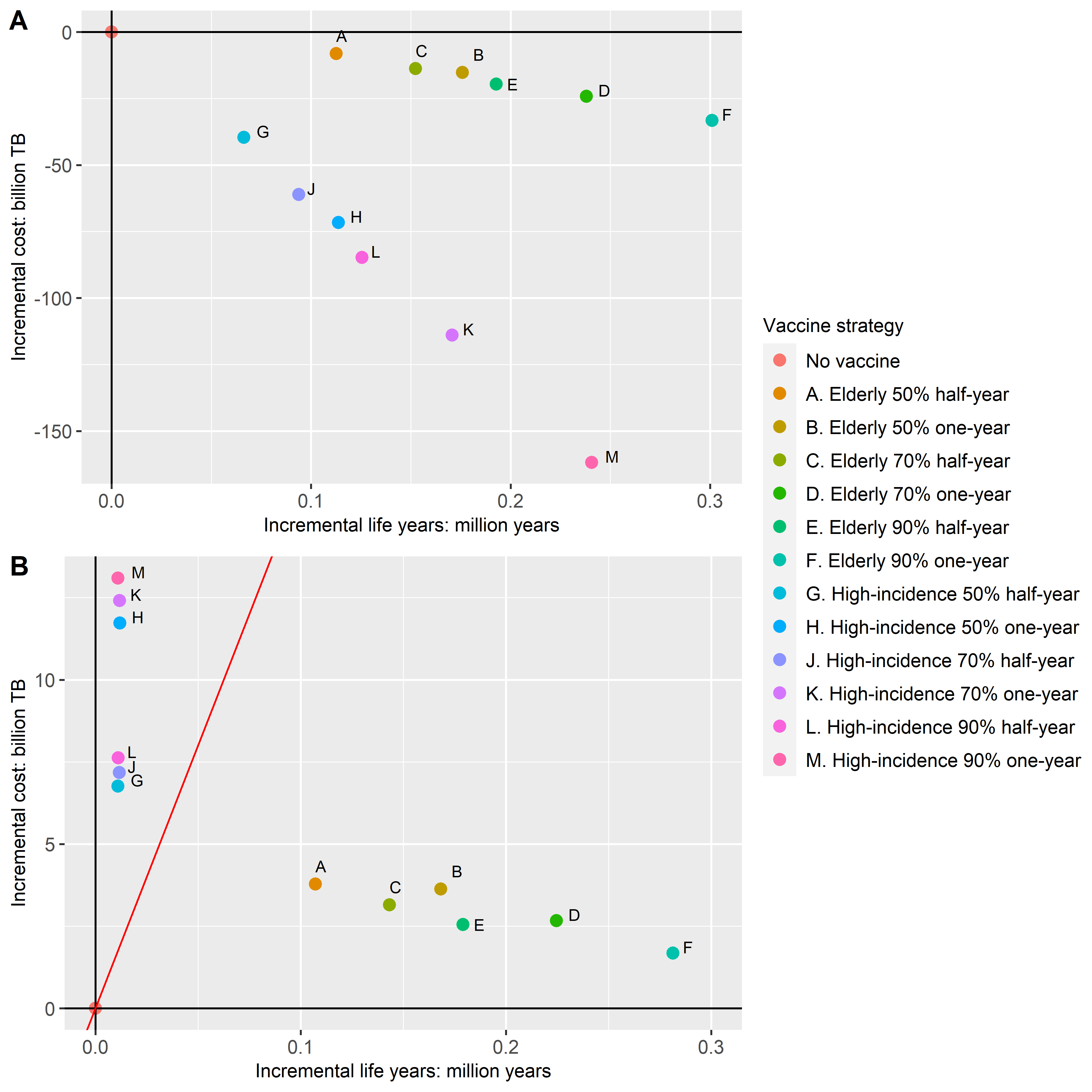
**

Notes: Panel A considers a vaccine that blocks infection. Panel B considers a vaccine that reduce severity. The lines in the Figure represent the 160,000 Thai Baht per life-year. There are two groups: high-risk elderly and high-incidence group aged 20-39 years old. Efficacy can be at 50%, 70%, and 90%, while duration of protection is at half-year or one year.

**Fig. S6**  **Incremental NMB versus CET for vaccines blocking infection and reducing severity**


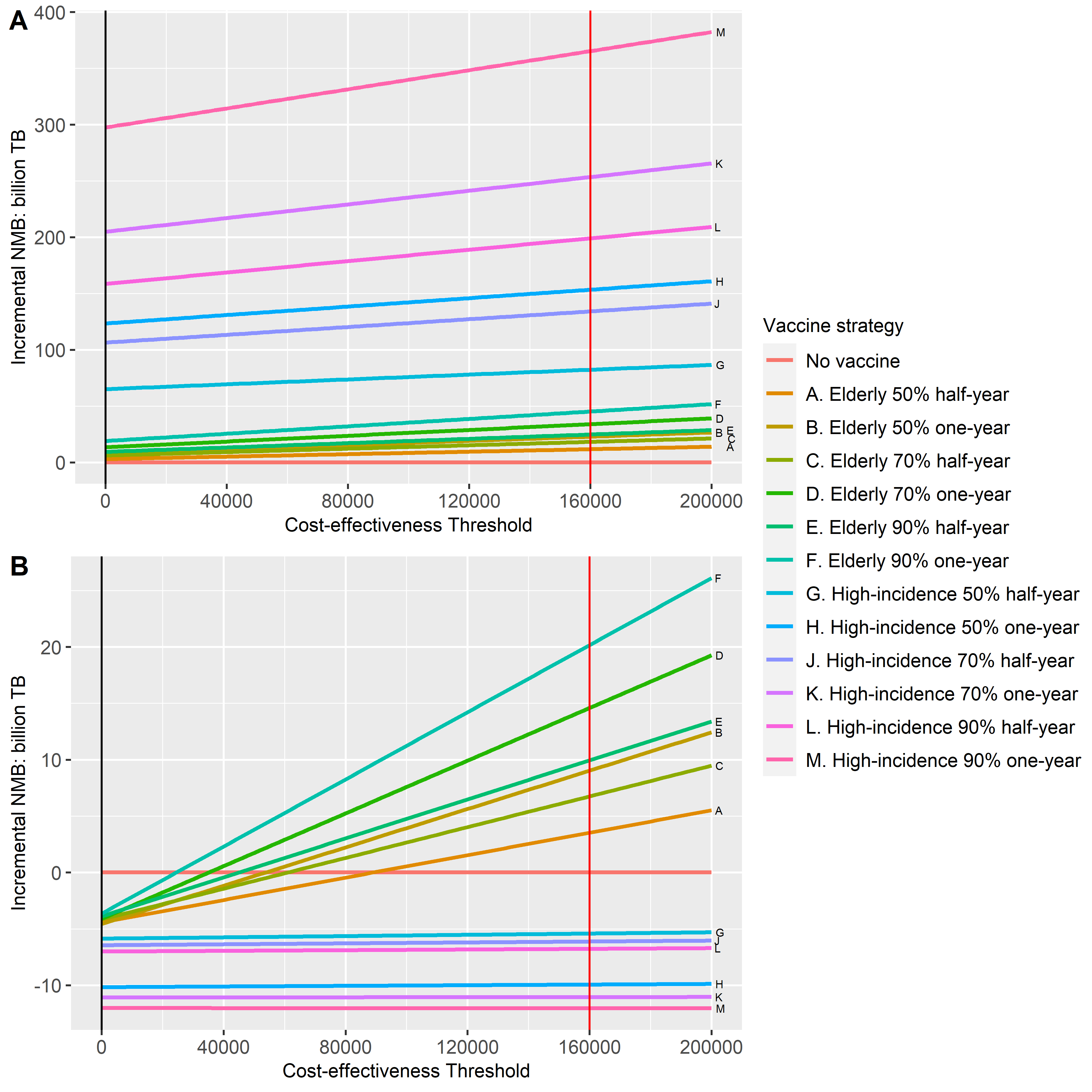


Notes: Panel A considers a vaccine that blocks infection. Panel B considers a vaccine that reduce severity.

**Supplementary Information 4**

**Table S2. Breakdown Cost Components**

| **Scenario** | **Dmed** | **Mask** | **Hygiene** | **Vaccine.aqui** | **Vaccine.supchain** | **Vaccine.admin** | **Adv.Event** | **CT** | **Quarantine** | **SD** | **Total** |
| --- | --- | --- | --- | --- | --- | --- | --- | --- | --- | --- | --- |
| Baseline_w_sd_40_rv1 | 107.02 | 7.65 | 25.15 | 0.00 | 0.00 | 0.00 | 0.0000 | 51.16 | 384.48 | 45.51 | 620.98 |
| Eff1_5M_AgeAdu_70_sd40_rv1 | 93.80 | 7.65 | 25.15 | 0.93 | 0.20 | 1.24 | 0.0007 | 44.99 | 338.12 | 45.51 | 557.60 |
| Eff1_5M_AgeEld_70_sd40_rv1 | 104.65 | 7.65 | 25.15 | 0.93 | 0.20 | 1.24 | 0.0007 | 50.25 | 377.67 | 45.51 | 613.26 |
| Eff1_5M_AgeHig_70_sd40_rv1 | 87.60 | 7.65 | 25.15 | 0.93 | 0.20 | 1.24 | 0.0007 | 41.93 | 315.11 | 45.51 | 525.32 |
| Eff1_09M_AgeAdu_70_sd40_rv1 | 80.51 | 7.65 | 25.16 | 1.67 | 0.35 | 2.23 | 0.0013 | 38.72 | 291.01 | 45.51 | 492.82 |
| Eff1_09M_AgeEld_70_sd40_rv1 | 102.89 | 7.65 | 25.15 | 1.67 | 0.35 | 2.23 | 0.0013 | 49.57 | 372.59 | 45.51 | 607.62 |
| Eff1_09M_AgeHig_70_sd40_rv1 | 65.64 | 7.65 | 25.16 | 1.67 | 0.35 | 2.23 | 0.0013 | 31.46 | 236.44 | 45.51 | 416.12 |
| Eff1_15M_AgeAdu_70_sd40_rv1 | 52.34 | 7.65 | 25.16 | 2.79 | 0.59 | 3.72 | 0.0022 | 25.29 | 190.06 | 45.51 | 353.11 |
| Eff1_15M_AgeHig_70_sd40_rv1 | 26.70 | 7.65 | 25.16 | 2.79 | 0.59 | 3.72 | 0.0022 | 12.83 | 96.41 | 45.51 | 221.36 |
| Eff3_5M_AgeAdu_70_sd40_rv1 | 107.42 | 7.65 | 25.15 | 0.93 | 0.20 | 1.24 | 0.0007 | 51.46 | 386.75 | 45.51 | 626.31 |
| Eff3_5M_AgeEld_70_sd40_rv1 | 106.84 | 7.65 | 25.15 | 0.93 | 0.20 | 1.24 | 0.0007 | 51.17 | 384.57 | 45.51 | 623.26 |
| Eff3_5M_AgeHig_70_sd40_rv1 | 107.57 | 7.65 | 25.15 | 0.93 | 0.20 | 1.24 | 0.0007 | 51.54 | 387.37 | 45.51 | 627.16 |
| Eff3_09M_AgeAdu_70_sd40_rv1 | 107.74 | 7.65 | 25.15 | 1.67 | 0.35 | 2.23 | 0.0013 | 51.70 | 388.56 | 45.51 | 630.57 |
| Eff3_09M_AgeEld_70_sd40_rv1 | 106.70 | 7.65 | 25.15 | 1.67 | 0.35 | 2.23 | 0.0013 | 51.18 | 384.64 | 45.51 | 625.09 |
| Eff3_09M_AgeHig_70_sd40_rv1 | 108.00 | 7.65 | 25.15 | 1.67 | 0.35 | 2.23 | 0.0013 | 51.84 | 389.65 | 45.51 | 632.07 |
| Eff3_15M_AgeAdu_70_sd40_rv1 | 108.22 | 7.65 | 25.15 | 2.79 | 0.59 | 3.72 | 0.0022 | 52.06 | 391.28 | 45.51 | 636.97 |
| Eff3_15M_AgeHig_70_sd40_rv1 | 108.63 | 7.65 | 25.15 | 2.79 | 0.59 | 3.72 | 0.0022 | 52.29 | 393.05 | 45.51 | 639.39 |
| Baseline_w_sd_00_rv1 | 151.19 | 7.65 | 25.15 | 0.00 | 0.00 | 0.00 | 0.0000 | 72.35 | 543.79 | 0.00 | 800.13 |
| Eff1_5M_AgeAdu_70_sd0_rv1 | 141.12 | 7.65 | 25.15 | 0.93 | 0.20 | 1.24 | 0.0007 | 67.79 | 509.48 | 0.00 | 753.56 |
| Eff1_5M_AgeEld_70_sd0_rv1 | 147.28 | 7.65 | 25.15 | 0.93 | 0.20 | 1.24 | 0.0007 | 70.89 | 532.81 | 0.00 | 786.14 |
| Eff1_5M_AgeHig_70_sd0_rv1 | 137.90 | 7.65 | 25.15 | 0.93 | 0.20 | 1.24 | 0.0007 | 66.08 | 496.68 | 0.00 | 735.83 |
| Eff1_09M_AgeAdu_70_sd0_rv1 | 133.46 | 7.65 | 25.15 | 1.67 | 0.35 | 2.23 | 0.0013 | 64.30 | 483.27 | 0.00 | 718.09 |
| Eff1_09M_AgeEld_70_sd0_rv1 | 144.39 | 7.65 | 25.15 | 1.67 | 0.35 | 2.23 | 0.0013 | 69.81 | 524.71 | 0.00 | 775.97 |
| Eff1_09M_AgeHig_70_sd0_rv1 | 127.64 | 7.65 | 25.15 | 1.67 | 0.35 | 2.23 | 0.0013 | 61.24 | 460.27 | 0.00 | 686.22 |
| Eff1_15M_AgeAdu_70_sd0_rv1 | 122.38 | 7.65 | 25.15 | 2.79 | 0.59 | 3.72 | 0.0022 | 59.21 | 445.02 | 0.00 | 666.51 |
| Eff1_15M_AgeHig_70_sd0_rv1 | 111.49 | 7.65 | 25.15 | 2.79 | 0.59 | 3.72 | 0.0022 | 53.60 | 402.82 | 0.00 | 607.81 |
| Eff3_5M_AgeAdu_70_sd0_rv1 | 151.54 | 7.65 | 25.15 | 0.93 | 0.20 | 1.24 | 0.0007 | 72.70 | 546.37 | 0.00 | 805.77 |
| Eff3_5M_AgeEld_70_sd0_rv1 | 150.70 | 7.65 | 25.15 | 0.93 | 0.20 | 1.24 | 0.0007 | 72.30 | 543.43 | 0.00 | 801.61 |
| Eff3_5M_AgeHig_70_sd0_rv1 | 151.75 | 7.65 | 25.15 | 0.93 | 0.20 | 1.24 | 0.0007 | 72.81 | 547.25 | 0.00 | 806.98 |
| Eff3_09M_AgeAdu_70_sd0_rv1 | 151.83 | 7.65 | 25.15 | 1.67 | 0.35 | 2.23 | 0.0013 | 72.98 | 548.48 | 0.00 | 810.34 |
| Eff3_09M_AgeEld_70_sd0_rv1 | 150.33 | 7.65 | 25.15 | 1.67 | 0.35 | 2.23 | 0.0013 | 72.27 | 543.14 | 0.00 | 802.80 |
| Eff3_09M_AgeHig_70_sd0_rv1 | 152.22 | 7.65 | 25.15 | 1.67 | 0.35 | 2.23 | 0.0013 | 73.19 | 550.08 | 0.00 | 812.54 |
| Eff3_15M_AgeAdu_70_sd0_rv1 | 152.28 | 7.65 | 25.15 | 2.79 | 0.59 | 3.72 | 0.0022 | 73.41 | 551.73 | 0.00 | 817.33 |
| Eff3_15M_AgeHig_70_sd0_rv1 | 152.94 | 7.65 | 25.15 | 2.79 | 0.59 | 3.72 | 0.0022 | 73.76 | 554.40 | 0.00 | 821.01 |

Notes: this table shows the breakdown cost for each component. All cost were converted into 2020 Thai Baht.

Dmed: direct medical csot; Vaccine.aqui: cost of vaccine acquisition; Vaccine.supchain: cost of vaccine supply chain; Vaccine.admin: cost of vaccine administration; Adv.Event: direct medical cost due to vaccine-related adverse event; CT: contract tracing; SD: social distancing.

Baseline_w_sd_40_rv1: no vaccine and with social distancing

Baseline_w_sd_00_rv1: no vaccine and without social distancing

Scenarios start with Eff: vaccine efficacy type + number of people being vaccinated + population group to vaccinate + vaccine efficacy + with/without social distancing + protection duration

- vaccine efficacy type: Eff1 – infection blocking; Eff3 – severity reduction
- number of people being vaccinated: 5M – 5 million; 09M – 9 million; 15M – 15 million
- population group to vaccinate: AgeAdu: adult group; AgeEld: elderly group; AgeHig: high-incidence group
- vaccine efficacy: 70 – 70%
- with/without social distancing: sd40 – with social distancing; sd0 – without social distancing
- protection duration: rv1 – 1 year

**Supplementary Information 5**

**Table S3. Breakeven Cost of Social Distancing – Equal Cost between Strategies with and without Social Distancing**

|  | **Percentage of GDP** | **Absolute value (Billion THB)** |
| --- | --- | --- |
| Baseline | 1.80% | 288.68 |
| Eff1_5M_AgeAdu_70_rv1 | 2.00% | 306.68 |
| Eff1_5M_AgeEld_70_rv1 | 1.70% | 272.25 |
| Eff1_5M_AgeHig_70_rv1 | 2.10% | 327.36 |
| Eff1_09M_AgeAdu_70_rv1 | 2.20% | 341.18 |
| Eff1_09M_AgeEld_70_rv1 | 1.70% | 260.34 |
| Eff1_09M_AgeHig_70_rv1 | 2.60% | 400.89 |
| Eff1_15M_AgeAdu_70_rv1 | 2.80% | 444.01 |
| Eff1_15M_AgeHig_70_rv1 | 3.40% | 534.38 |
| Eff3_5M_AgeAdu_70_rv1 | 1.80% | 285.37 |
| Eff3_5M_AgeEld_70_rv1 | 1.80% | 278.11 |
| Eff3_5M_AgeHig_70_rv1 | 1.80% | 288.3 |
| Eff3_09M_AgeAdu_70_rv1 | 1.80% | 282.64 |
| Eff3_09M_AgeEld_70_rv1 | 1.70% | 270 |
| Eff3_09M_AgeHig_70_rv1 | 1.80% | 288.17 |
| Eff3_15M_AgeAdu_70_rv1 | 1.80% | 278.89 |
| Eff3_15M_AgeHig_70_rv1 | 1.80% | 288.47 |

Notes:

Baseline: no vaccine

Scenarios start with Eff: vaccine efficacy type + number of people being vaccinated + population group to vaccinate + vaccine efficacy + + protection duration

- vaccine efficacy type: Eff1 – infection blocking; Eff3 – severity reduction
- number of people being vaccinated: 5M – 5 million; 09M – 9 million; 15M – 15 million
- population group to vaccinate: AgeAdu: adult group; AgeEld: elderly group; AgeHig: high-incidence group
- vaccine efficacy: 70 – 70%
- protection duration: rv1 – 1 year

**Supplementary Information 6**

**Time series analysis: The impact of social distancing on the economy**

**METHODOLOGY**

Our main objective is to determine the reduction change in GDP with NPI implementation. However, we cannot run regression directly between GDP and NPI due to the difference in frequency of data in published reports. Accordingly, we examined two hypotheses to determine whether we can use the stock market index as an intermediate outcome to link the effects of NPIs to GDP (*Figure S3*). First hypothesis is that GDP and stock market index has a positive association. Secondly, NPIs can negatively impact the stock market index through direct and indirect channels.


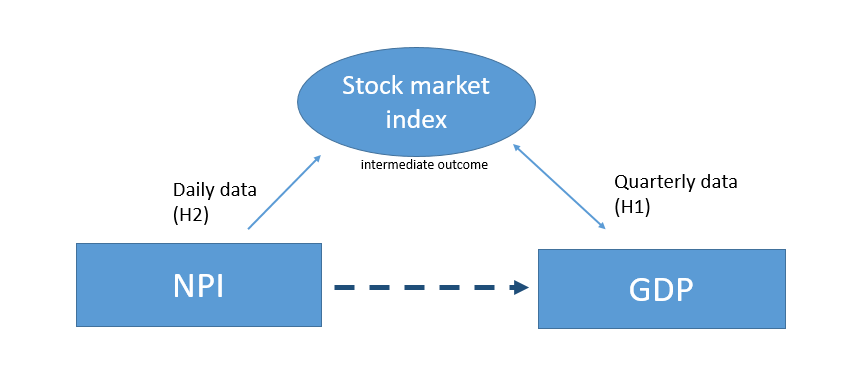


*Fig S6*. Linking NPI to GDP through stock market index

**1. Hypothesis 1: GDP and Stock market index**

We determined association between GDP and stock market index through Ordinary least squares (OLS) method using the first difference (14). We used quarterly data for nominal GDP and stock market index (average closing values) from March 2000 to June 2020 for the analysis. We also accounted for quarterly seasonality using dummy variables (*Equation 1*).

${d1GDP}_{t}=\beta o+{\beta1d1stocks}_{t-1}+\beta2Q1+\beta3Q2+\beta4Q3+\beta5Q4+\varepsilon1$ [Equation 1]

where *GDP* is real GDP to adjust for inflation and *stocks* is the mean stock market index for the quarter

**2. Hypothesis 2: Stock market index and NPIs**

*Dataset*

Daily stock market index data was collected from Nov. 2019 to Nov. 2020 to estimate differences in pre/post-intervention trends. However, given the difficulty to separate the individual effects of NPIs as they were implemented simultaneously, we combined the Oxford COVID-19 Government Response Tracker (OxCGRT) policy indicators, recoded them as binary variables, and then redefined NPIs as (1) social distancing – school, work closures, and limits on gatherings; (2) border closure – quarantine/ ban arrivals from some or all regions (15). We also revised the dataset based on their announcement dates.

Stock market index may not only be influenced by NPIs but also by other factors that can affect investor’s confidence and pandemic perception. For this reason, the following control variables were considered in the model:

(a) *COVID-19 related variables such as the daily number of global and local COVID-19* (16). The indirect impact of these two control variables was incorporated using interaction terms. Moreover, weekly local news trends from Google trends on ‘coronavirus’ were also considered (Google trend, 2020).

(b) *Dow Jones Index, oil and gold price* (Yahoo Finance, 2020) were incorporated as continuous variables to control how the local stock market reacts to the global economic status.

(c) Lastly, fiscal stimulus package was also added through a binary variable (OxCGRT, Hale, et al., 2021)

Model

For the second hypothesis, we use ARIMA models to allow the inclusion of lagged variables to incorporate feedback over time, and to obtain unbiased estimates (17) (*Equation 2&3*),

${SET}_{t}=\beta o+\beta_{1}socialdis_{t}+\beta_{2}borderclose_{t}+\gamma{control}_{t}+\epsilon_{t}$ [Equation 2]

$\epsilon_{t}= \sum_{j=1}^{p} \rho_{j}\epsilon_{t-j}+ \sum_{k=1}^{q} q_{k}\mu_{t-k}+\mu_{t}$

where *SET* is the stock market index closing value, and social distancing (*socialdis*) and border closure (*borderclose*) are the NPIs of interest. The term *control* represents the aggregate of the control variables considered in the model – local and global COVID-19 cases, coronavirus news trend, Dow Jow Index, oil price, gold price, fiscal stimulus. Lastly, the parameter $\epsilon_{t}$ represents a polynomial of autoregressive (AR) and moving average (MA) parameters of the fitted model.

In the time series model, the order of AR and MA terms were identified through the goodness of fit and parsimonious criteria (Akaike information criterion and Bayesian information criterion) (17).

**3. Computing magnitude of the effect of NPI on the GDP**

Given the structural uncertainty surrounding the models for Hypothesis 2, we chose five plausible models – AR (0), AR (1), AR (2), AR (1) MA (1), AR (1) MA (2) – to calculate the impact of NPI on the GDP. To compute the magnitude of the effect of NPI on the GDP, we multiplied the coefficient of NPIs for each model to coefficient of SET from Equation 1 to estimate the real GDP for the quarter. As we also used original values and transformed the dataset to their natural logarithmic values, we performed certain adjustments to obtain the estimated annual GDP percentage reduction from the NPI. The resulting percentage values were then multiplied to the nominal GDPs to derive the impact in absolute values.

**References**

1. Linton NM, Kobayashi T, Yang Y, Hayashi K, Akhmetzhanov AR, Jung S-m, et al. Incubation period and other epidemiological characteristics of 2019 novel coronavirus infections with right truncation: a statistical analysis of publicly available case data. J Clin Med. 2020;9(2):538.

2. Khalili M, Karamouzian M, Nasiri N, Javadi S, Mirzazadeh A, Sharifi H. Epidemiological characteristics of COVID-19: a systematic review and meta-analysis. Plos One. 2020;148.

3. Aguas R, White L, Hupert N, Shretta R, Pan-Ngum W, Celhay O, et al. Modelling the COVID-19 pandemic in context: an international participatory approach. BMJ global health. 2020;5(12):e003126.

4. United Nations. Revision of world population prospects. 2019.

5. Prem K, Cook AR, Jit M. Projecting social contact matrices in 152 countries using contact surveys and demographic data. PLoS Comput Biol 2017;13(9):e1005697.

6. Department of Disease Control of Thailand. COVID-19: Thailand Situation: Ministry of Public Health; 2021 [Available from: <https://ddc.moph.go.th/viralpneumonia/eng/index.php>.

7. Nishiura H, Kobayashi T, Miyama T, Suzuki A, Jung S-m, Hayashi K, et al. Estimation of the asymptomatic ratio of novel coronavirus infections (COVID-19). Int J Infect Dis. 2020;94:154.

8. Alene M, Yismaw L, Assemie MA, Ketema DB, Mengist B, Kassie B, et al. Magnitude of asymptomatic COVID-19 cases throughout the course of infection: A systematic review and meta-analysis. PloS one. 2021;16(3):e0249090.

9. Yanes-Lane M, Winters N, Fregonese F, Bastos M, Perlman-Arrow S, Campbell JR, et al. Proportion of asymptomatic infection among COVID-19 positive persons and their transmission potential: A systematic review and meta-analysis. PloS one. 2020;15(11):e0241536.

10. Oran DP, Topol EJ. The proportion of SARS-CoV-2 infections that are asymptomatic: a systematic review. Annals of internal medicine. 2021;174(5):655-62.

11. Petrilli CM, Jones SA, Yang J, Rajagopalan H, O'Donnell LF, Chernyak Y, et al. Factors associated with hospitalization and critical illness among 4,103 patients with COVID-19 disease in New York City. MedRxiv. 2020.

12. Petrilli CM, Jones SA, Yang J, Rajagopalan H, O’Donnell L, Chernyak Y, et al. Factors associated with hospital admission and critical illness among 5279 people with coronavirus disease 2019 in New York City: prospective cohort study. bmj. 2020;369.

13. Chang R, Elhusseiny KM, Yeh Y-C, Sun W-Z. COVID-19 ICU and mechanical ventilation patient characteristics and outcomes—A systematic review and meta-analysis. PloS one. 2021;16(2):e0246318.

14. Shrestha MB, Bhatta GR. Selecting appropriate methodological framework for time series data analysis. The Journal of Finance and Data Science. 2018;4(2):71-89.

15. Hale T, Angrist N, Goldszmidt R, Kira B, Petherick A, Phillips T, et al. A global panel database of pandemic policies (Oxford COVID-19 Government Response Tracker). Nat Hum Behav. 2021.

16. Coronavirus Pandemic (COVID-19 [Internet]. OurWorldInData.org. 2020. Available from: <https://ourworldindata.org/coronavirus>.

17. Cuadrado C, Dunstan J, Silva-Illanes N, Mirelman AJ, Nakamura R, Suhrcke M. Effects of a sugar-sweetened beverage tax on prices and affordability of soft drinks in Chile: A time series analysis. Social Science & Medicine. 2020;245:112708.
